# Investigation of a viable but non-culturable state in *Porphyromonas gingivalis* and host cell invasion

**DOI:** 10.1371/journal.pone.0340605

**Published:** 2026-01-16

**Authors:** Adenrele Oludiran, Benjamin Lewis, Cole Pudwill, Sasanka Chukkapalli, Hanie Ahmadi, Daria Bannova, Alexander Linares, Jacob Burks, Jeffrey D. Hillman, William A. Dunn, Ann Progulske-Fox

**Affiliations:** 1 Center for Molecular Microbiology, University of Florida, Gainesville, Florida, United States of America; 2 Department of Biomedical Engineering, Texas A&M University, College Station, Texas, United States of America; 3 Department of Anatomy and Cell Biology, University of Florida College of Medicine, Gainesville, Florida, United States of America; Loma Linda University Health School of Medicine, UNITED STATES OF AMERICA

## Abstract

*Porphyromonas gingivalis* (*P. gingivalis*) is a gram-negative, black-pigmented, anaerobic pathogen known for its biofilm formation and its central role in periodontal disease. More recently, *P. gingivalis* has been implicated in various systemic conditions, including atherosclerosis, Alzheimer’s disease, and certain types of cancer, such as pancreatic and oral cancer. This bacterium employs several mechanisms to evade environmental stress, thereby contributing to its pathogenicity. The viable but non-culturable (VBNC) state is characterized by bacteria that remain viable but have reduced metabolic activity and are unable to form colonies on conventional culture media. To induce the VBNC state in *P. gingivalis*, we subjected the bacterium to oxidative stress using H_2_O_2_ and subsequently resuscitated it from this state with sodium pyruvate. We utilized viability staining, confocal microscopy, and flow cytometry (FC) to count live and dead bacteria, confirming the presence of significant numbers of viable *P. gingivalis* cells both before and after stress induction. Despite being viable, the stressed *P. gingivalis* failed to form colonies on blood agar plates after seven days of incubation, indicating it had entered the VBNC state. We were then able to resuscitate the VBNC *P. gingivalis* by adding sodium pyruvate, and the growth of the resuscitated bacteria on plates was comparable to that of control *P. gingivalis*. Investigation into the invasiveness of *P. gingivalis* in the VBNC state was conducted using human coronary artery endothelial cells (HCAECs). *P. gingivalis* in the VBNC state demonstrated the ability to invade and based on live/dead staining, showed that a substantial proportion of the VBNC *P. gingivalis* remained viable within the host cells for extended periods. In this study, we explore the VBNC survival strategy previously described in many aerobic bacteria but not previously reported in anaerobes such as *P. gingivalis*. The objectives of this study are to verify the VBNC state in *P. gingivalis,* determine whether this state can be reversed and assess the extent to which it impacts the ability of *P. gingivalis* to invade host cells. Understanding the VBNC and resuscitation states will be instrumental in guiding the development of more effective therapies for periodontitis and other diseases associated with *P. gingivalis* infection.

## Introduction

The viable but non-culturable (VBNC) state is a survival strategy employed by many bacteria in response to environmental stress. In this state, bacteria remain alive with reduced metabolic activity but lose the ability to grow on routine laboratory bacteriological media plates or broth [1]. The VBNC phenomenon was first identified in *Escherichia coli* and *Vibrio cholerae* in 1982 [2,3]. Since then, it has been observed in a wide range of aerobic and facultative anaerobic bacterial species, but it has not yet been reported in any obligate anaerobic bacterial species. Environmental stressors that induce the VBNC state are often species-specific, but some are commonly shared by many bacterial species, including oxidative stress, starvation, pH changes, temperature changes, and the presence of antimicrobial agents [1,4,5]. Induction of the VBNC state is characterized by a reduction in metabolic activity, which is presumed to increase the chance of survival by bacteria in a hostile environment [6]. Notably, oxidative and antibiotic stresses are of particular interest, as they are either natural components of the host immune response or are introduced therapeutically to combat bacterial infections [2]. One of the components of the innate immune system, polymorphonuclear neutrophils (PMNs), usually involved in the active killing of bacteria in humans and other animals, was reported to induce a VBNC state in a subpopulation of the bacterium *Listeria monocytogenes* enabling it to persist intravascularly *ex vivo* [7].

*Porphyromonas gingivalis,* a gram-negative, black-pigmented, anaerobic pathogen, is recognized as one of the primary causative agents of periodontal disease. It is also increasingly associated with a variety of systemic diseases including diabetes, cardiovascular disease, preterm birth, Alzheimer’s disease [8,9] and oral cancer [10]. Bahar *et al*. (2021) demonstrated a direct link between infection with *P. gingivalis* and Alzheimer’s disease in a wild-type obese and diabetic mouse model [8]. For years, researchers failed to culture *P. gingivalis* from atherosclerotic tissues, despite detecting its DNA. Progulske-Fox et al. [11] were the first to demonstrate viable *P. gingivalis* in human vascular cells taken from diseased tissue samples, showing that it could survive intracellularly without being culturable. Later work using a laboratory model showed that *P. gingivalis* loses culturability after 48 hours while inside host cells but can regain culturability when the infected host cells are lysed and the lysate containing unculturable *P. gingivalis* is allowed to reinfect new host cells [12]. These findings have helped to broaden our understanding of *P. gingivalis’s* potential ability to cause extra-oral diseases. Based on these findings, we hypothesized that *P. gingivalis* can enter a semi-dormant state, specifically, the VBNC state, which may enable it to survive and evade host defenses during systemic dissemination [13–15].

In this study, we investigated the ability of *P. gingivalis* strain W83 to enter a VBNC state, resuscitate from this state, as well as invade human coronary artery endothelial cells (HCAEC). Additionally, we found that prolonged incubation of *P. gingivalis* within HCAEC primary cells can induce the VBNC state, possibly through oxidative stress. Understanding the VBNC state in *P. gingivalis* is not only critical for comprehending its role in periodontal disease but also for addressing its broader implications in systemic health. As research continues to uncover the connections between oral pathogens and non-oral diseases, the need to address bacterial persistence mechanisms, such as the VBNC state, becomes increasingly important.

## Materials and methods

### Bacterial growth conditions

*P. gingivalis* strain W83 was used throughout these studies. Colonies were grown on blood agar plates (BAPs; 1.5% tryptic soy agar, 5% defibrinated sheep blood, supplemented with 5 μg/mL hemin and 1 μg/mL vitamin K) containing 50 μg/mL gentamycin. Liquid cultures were prepared in antibiotic-free tryptic soy broth medium (TSB; supplemented with 5 μg/mL hemin and 1 μg/mL vitamin K) by inoculation with black colonies of *P. gingivalis* from the BAPs. Both agar and liquid cultures were incubated in an anaerobic chamber at 37°C under conditions of 85% N_2_, 10% H_2_, and 5% CO_2_ (COY Lab Products). Optical density (OD) measurements were conducted using both a BIO-RAD SmartSpec™ Plus spectrophotometer at OD_550_ and a Biowave CO_800_ Cell at OD_600_.

### VBNC induction, resuscitation, and viability staining

Liquid cultures of *P. gingivalis* were grown overnight in supplemented TSB medium as stated in the bacterial growth and condition section. The overnight culture was subcultured to 0.2 OD_600_ in fresh TSB media and allowed to grow to 0.8 OD_600_ under anaerobic conditions. To induce the VBNC state, 70% of the culture was centrifuged at 4,000xg for 15 minutes and the pellet was resuspended in TSB medium containing 3 mM hydrogen peroxide (H_2_O_2_). The resuspended culture was then gently vortexed and left undisturbed at 37°C under anaerobic conditions for 30 minutes, after which catalase was added to a final concentration of 100 μg/mL and allowed to incubate at 37°C for 10 minutes to assure complete elimination of H_2_O_2_. For resuscitation, half of the H_2_O_2_ -treated culture was centrifuged. The pellet was resuspended in TSB media containing 1 mM sodium pyruvate and allowed to incubate anaerobically at 37°C under anaerobic conditions for 90 minutes. Thirty percent (30%) of the remaining original culture served as a control, undergoing the same steps but without the additions of H_2_O_2_, catalase, and pyruvate. At the end of their respective treatments, serially diluted samples from each condition were plated on BAPs containing 50 μg/mL gentamicin for CFU/mL enumeration following 7 days of anaerobic incubation. Bacterial viability assays were also performed on 1 ml of samples from each of the experimental groups using the BacLight LIVE/DEAD bacterial viability kit (Invitrogen) [5], followed by imaging with a confocal microscope.

#### Flow cytometry bacterial count.

The live/dead assay protocol was adapted for flow cytometry using the three treatment conditions: Control *P. gingivalis* (NT, not treated), H_2_O_2_-treated *P. gingivalis* (VBNC), and H_2_O_2_- and pyruvate-treated *P. gingivalis* (Resuscitated). Control *P. gingivalis* was divided into four samples: Control a (no stain control), Control b (45 minutes, 90°C heat-killed, stained with propidium iodide (PI, red) alone, Control c (stained with SYTO 9, green) alone, and Control d (double-stained with PI and SYTO 9). VBNC state *P. gingivalis* and Resuscitated *P. gingivalis* samples were double stained with PI and SYTO 9. All 1mL samples were diluted 1:4 in 0.8% NaCl solution and filtered (Allpure Biotechnology nylon filter, 5.0µm PORE size) to separate intact bacteria from cellular debris. The filtrate containing intact bacteria was kept on ice before subjecting it to analysis using the Attune Nxt Acoustic Focusing Cytometer (Fisher) with proper compensation [16,17].

#### Mammalian cell culture.

Human coronary artery endothelial cells (HCAEC) (Lonza) were cultured at passage 5 and maintained in microvascular endothelial growth medium-2 (EGM-2 MV) medium (Lonza) supplemented with 5% FBS, 0.04% hydrocortisone, 4% hFGF-B, 0.1% VEGF, 0.1% R3-IGF-1, 0.1% ascorbic acid, and 0.1% hEGF, with or without GA-1000 (Gentamicin sulfate and Amphotericin-B, 0.33%). The cells were grown in a water jacketed incubator at 37°C and with added 5% CO_2_.

#### VBNC infection assay.

HCAEC were seeded at a density of 1x10^5^ cells/mL in 12-well plates with GA-1000-containing EGM-2 MV medium and after 24 hours of incubation the medium was replaced with fresh antibiotic-containing medium. When the HCAEC reached 70% confluency, the endothelial cell invasion assay was conducted according to the following invasion protocol: HCAEC were infected under sterile, aerobic conditions with *P. gingivalis* at a multiplicity of infection (MOI) of 100 bacteria per HCAEC cell based on a standard curve of CFU versus OD_600_. *P. gingivalis* samples used in the invasion assay included control cultures, H_2_O_2-_treated (VBNC) cultures, and H_2_O_2_- and pyruvate-treated (Resuscitated) cultures, each at approximately an OD_600_ of 0.8. Serial dilutions of each culture were also plated on BAPs before infection to determine culturable cell numbers. HCAEC infection experiments were conducted under aerobic conditions, then incubated in a humidified water-jacketed incubator at 37°C in air supplemented with 5% CO₂. Three wells in each 12-well plate containing HCAECs were reserved as negative controls (not infected with *P. gingivalis*). The infected and control HCAEC cells were incubated for 6, 24, 48, or 72 hours.

#### Mitotracker staining.

HCAECs cells were infected with control, VBNC-state, or resuscitated *P. gingivalis* at an MOI of 100. Three hours after infection, 300 μg/mL gentamicin and 400 μg/mL metronidazole were added to each HCAEC well to eliminate extracellular bacteria. The plates were then incubated for 6, 24, 48, or 72 hours. One hour before each time point, HCAEC cells from each group were washed with PBS, stained with 25 nM Mitotracker Red CMXRos (Invitrogen), and allowed to incubate in the dark for 45 minutes. After staining, cells were washed with PBS and fixed with 2% PFA at 4°C overnight. The cells were washed again and mounted on glass coverslips with VECTASHIELD Antifade Mounting Medium with 4’,6-diamidino-2-phenylindole (DAPI, VectorLabs) before imaging with a confocal microscope.

#### Intracellular CFU enumeration.

At 6-, 24-, 48-, and 72-hour time points, cells were centrifuged and washed with PBS, lysed with 1mL of ddH2O, and vortexed vigorously. The resulting 1mL of ddH_2_O lysate was serially diluted, and 100 µL samples were spread on BAPs for CFU enumeration [18,19].

#### Intracellular viability staining.

Cells were infected with control, VBNC-state, and Resuscitated *P. gingivalis* at an MOI of 75. Three hours after infection, each HCAEC well plate was treated with 300 μg/mL gentamicin and 400 μg/mL metronidazole. The plates were then incubated for 6, 24, 48, or 72 hours. At each time point, HCAEC cells were washed with a MOPS/MgCl_2_ solution and incubated with live/dead stains (2.5 μM SYTO 9 and 15 μM PI) in MOPS/MgCl_2_ containing 0.1% saponin for 10 minutes in the dark. After staining, cells were washed with MOPS/MgCl_2_ and imaged using a confocal microscope [18].

#### Confocal microscopy.

Imaging was performed using a Nikon Ti-2 E laser confocal microscope. Acquisition settings were optimized before imaging and kept consistent across all experiments. Laser power was maintained at or below 1.6 to minimize photobleaching, with scan averaging set to 4x. Images were obtained using a Plan Apo 20x dry objective at Nyquist resolution, calculated by NIS-Elements (Nikon) software.

#### Data analysis.

Statistical analyses were conducted using Prism (GraphPad Software, San Diego, CA, USA). ANOVA was used for all statistical analyses, with data expressed as means ± SEM. Differences were considered significant when p < 0.05, with the following significant levels: not significant (ns); *p < 0.05; **p < 0.01; **p < 0.001; ****p < 0.0001, respectively, as shown in the figures.

## Results

### The VBNC state is induced in *P. gingivalis* cultures in the presence of hydrogen peroxide and reversed after treatment with sodium pyruvate

**Fig 1:**
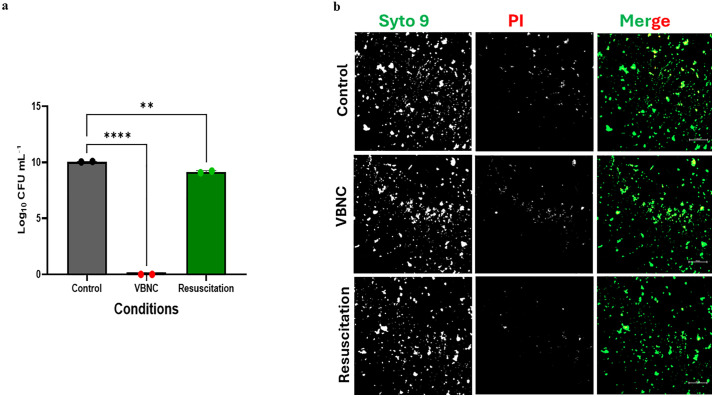
Culturability and viability of *P. gingivalis* cultures under three treatment conditions. **a)** Log plot of CFU/mL of *P. gingivalis* cultures that were untreated controls, treated with H_2_O_2_ (VBNC), or treated with H_2_O_2_ followed by incubation with sodium pyruvate (resuscitated); **b)** Confocal images of live/dead staining of the same three cultures; left and middle columns showing mono images of SYTO 9 and PI channels, respectively; right column shows color merge of SYTO 9 and PI channels. Scale bar = 100 μm.

Treatment with 3 mM H_2_O_2_ for 30 minutes followed by a 10-minute treatment with catalase to inactivate H₂O₂ caused a 10-log reduction in the number of viable colony forming cells as compared to the control (Fig 1). However, as shown in Fig 1b, confocal microscopy consistently showed the presence of viable cells (SYTO 9-labeled green fluorescence) in both control and H₂O₂ treated samples. Moreover, the merged SYTO 9/PI images qualitatively showed similar overall compositions of live and dead *P. gingivalis* cells in these two groups. These findings suggested the possibility that hydrogen peroxide treated cells had entered into a VBNC state. Treatment with H₂O₂ followed by 90-minute incubation in medium containing 1 mM sodium pyruvate resulted in a 9-log increase in the number of viable colony forming units relative to cells that were treated with just H₂O_2_ (Fig 1). Again, the presence of both live and dead cells in this group appeared qualitatively like the control and peroxide treated group (Fig 1b). These findings were interpreted to provide preliminary evidence that pyruvate could resuscitate VBNC cells. To provide a quantitative assessment of the similarities of live/dead cells in the three groups, five independent biological replicates of the merged images’ fluorescence were measured using ImageJ software for their SYTO9/PI ratio (Table 1). The ratios of live to dead cells were within a narrow range (1.1 to 1.7) for all three groups, confirming our impression that they appeared similar by visual examination.

**Table 1:** Quantified SYTO 9/PI fluorescent image measurement with determination of the ratio in five different biological samples.

Condition	Live SYTO9 (Mean ± SD)	Dead PI (Mean ± SD)	Ratio (SYTO9/PI)
**Control**	17.41 ± 14.74	13.32 ± 9.85	1.3
**VBNC**	15.09 ± 13.83	13.62 ± 7.98	1.1
**Resuscitation**	14.76 ± 7.05	8.57 ± 4.14	1.7

ImageJ software was used to measure fluorescence intensity from SYTO9 (live) and propidium iodide (PI; dead) signals in fluorescent images. The table presents the mean ± standard deviation (SD) of SYTO9 and PI intensities, along with the calculated SYTO9/PI ratio for each condition (control, VBNC, and pyruvate resuscitated cells).

### Flow cytometry confirms the viability of VBNC-state and resuscitated *P. gingivalis* bacteria

**Fig 2:**
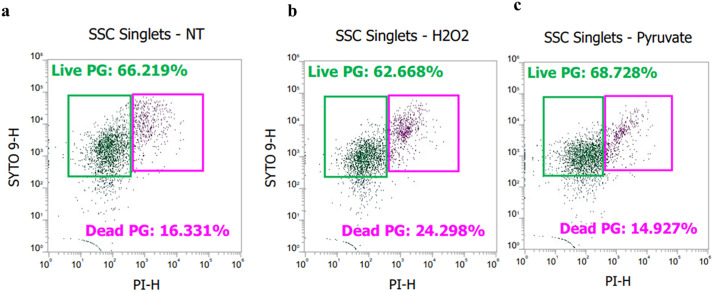
Flow cytometry counts of live and dead bacteria. Gates separate live (green) and dead (pink) bacterial cells from *P. gingivalis* cultures, which were **a)** control (NT), **b)** treated with H_2_O_2_, or **c)** treated with sodium pyruvate for 90 minutes after treatment with H_2_O_2_ (Pyruvate).

Flow cytometry (FC) was employed to quantify live and dead *P. gingivalis* cells from control, H_2_O_2_-treated, and H_2_O_2_ followed by pyruvate-treated cultures. The FC method, performed using a compensation protocol, provided quantification of viable and dead bacteria using the live/dead staining dyes (S1 Fig). The FC measures height, area, and stained fluorescence to show the results of the bacterial cells. The majority 66.225% of control *P. gingivalis* cells were found to be alive, with 16.331% dead; 62.67% of H_2_O_2_-treated *P. gingivalis* cells were found to be alive with 24.30% dead; and 68.73% of pyruvate-treated *P. gingivalis* cells were found to be alive with 14.93% dead (Fig 2). Thus, flow cytometry supported the findings from confocal live/dead images that most H_2_O_2_-treated *P. gingivalis* remained viable even though they were not culturable.

### *P.* *gingivalis* invasiveness and viability following host-cell invasion are independent of VBNC status

**Fig 3:**
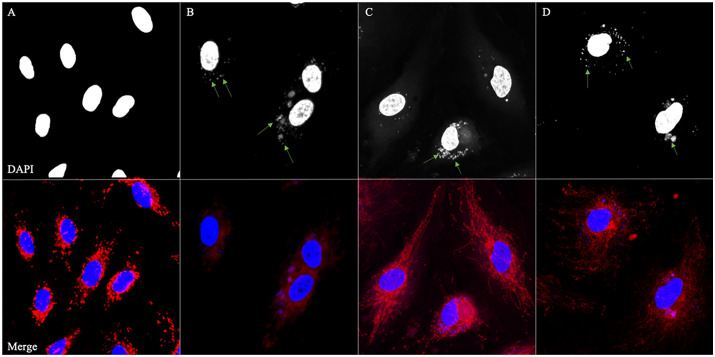
Invasion of HCAECs 6 hours post-infection by *P. gingivalis* in three different physiological states. Top: Mono images of DAPI staining of **A)** Uninfected HCAEC control **B)** HCAEC infected with control *P. gingivalis*, **C)** HCAEC infected with VBNC-state *P. gingivalis*, and **D)** HCAEC infected with resuscitated *P. gingivalis*. Bottom: Merged color images of DAPI staining (blue) with MitoTracker Red CMXRos (red) to highlight the HCAEC boundary. Green arrows highlight internalized *P. gingivalis*. Scale bar = 20 µm.

**Fig 4:**
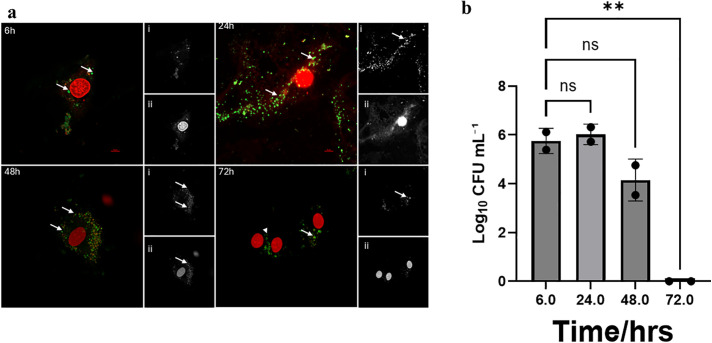
Micrographs of HCAEC infected with control *P. gingivalis* and colony counts of internalized bacteria. **(a)** Live/dead staining of HCAEC and internalized bacteria at 6-, 24-, 48-, and 72-hours post-infection; **(i)** SYTO 9 stained live *P. gingivalis* (green), **(ii)** PI-stained dead *P. gingivalis* (red). Scale bar= 10μm. **(b)** Log10 CFU/mL of invaded control (no treatment) -*P. gingivalis* at the same timepoints.

**Fig 5:**
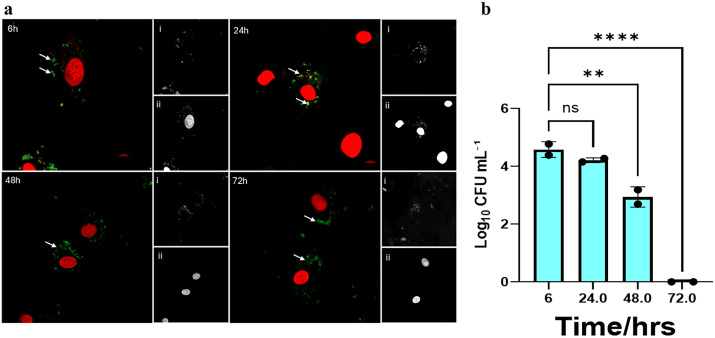
Micrographs of HCAEC infected with VBNC-state *P. gingivalis* and colony counts of internalized bacteria. **(a)** Live/dead staining of HCAEC and internalized bacteria at 6-, 24-, 48-, and 72-hours post-infection; **(i)** SYTO 9 stained live *P. gingivalis* (green), **(ii)** PI-stained dead *P. gingivalis* (red). Scale bar = 10μm. **(b)** Log_10_ CFU/mL of invaded VBNC-state *P. gingivalis* at the same time points.

**Fig 6:**
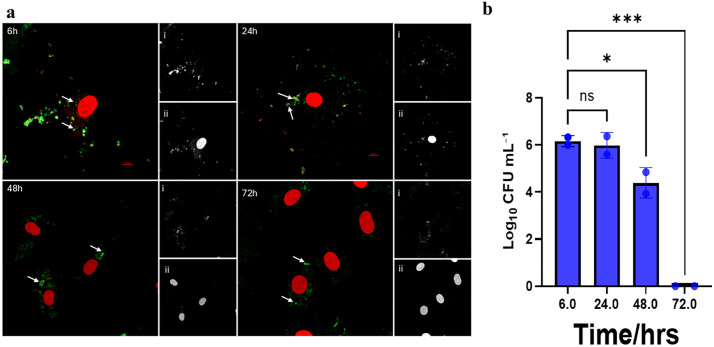
Micrographs of HCAEC infected with resuscitated *P. gingivalis* and colony counts of internalized bacteria. **a)** Live/dead staining of HCAEC and internalized bacteria at 6-, 24-, 48-, and 72-hours post-infection; **i)** SYTO 9 stained live *P. gingivalis* (green), **ii)** PI-stained dead *P. gingivalis* (red). Scale= 10μm. **b)** Log_10_ CFU/mL of invaded resuscitated *P. gingivalis* at the same time points.

We took the above results as strong evidence that, *in vitro,* an H_2_O_2_-induced oxidative stress can cause *P. gingivalis* to transition into a VBNC state and that exposure to sodium pyruvate can cause VBNC *P. gingivalis* to become resuscitated. To extend these findings, we infected HCAECs with *P. gingivalis* to assess its ability to invade host cells in control, VBNC, and resuscitated states. At 6, 24, 48, or 72 hours after HCAEC inoculation with *P. gingivalis* representing each of these three states, infected HCAECs were stained with DAPI (to highlight intracellular bacteria and host cell nuclei) and Mitotracker Red CMXRos (to highlight the intracellular space). At all tested time points, confocal microscope images showed all three physiological states of *P. gingivalis* appearing within their respective host-HCAECs as visible, blue-stained dots, indicating invasion into HCAECs. In uninfected control HCAECs, no intracellular bacteria were visible. Fig 3 shows the 6-hour time point, and the other time points are found in the supporting information figures (S2-S4 Fig). To assess the physiological state of *P. gingivalis* after the invasion, we performed intracellular live/dead viability assays. Confocal microscopy revealed the intracellular presence of both live and dead *P. gingivalis* in all three states at each time point (Figs 4a, 5a, and 6a). The uninfected control group showed only PI-stained HCAEC nuclei (S5 Fig).

#### The VBNC status of *P. gingivalis* is dynamic following host cell invasion observed in earlier studies.

**Fig 7:**
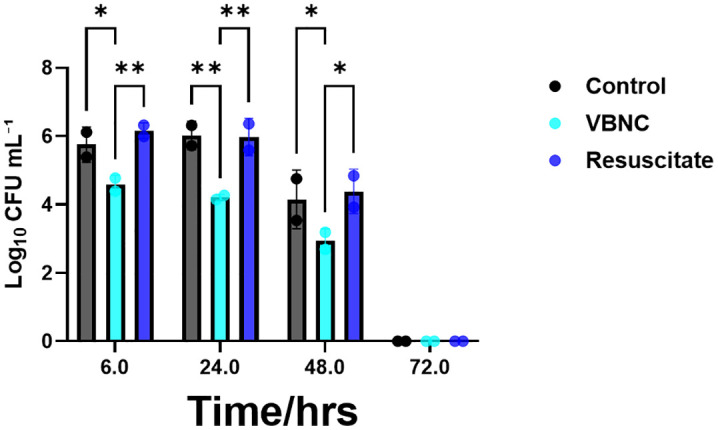
Invasion of HCAEC cells by *P. gingivalis* in the three experimental conditions over time. Recoverable CFUs of control *P. gingivalis*, VBNC *P. gingivalis*, and resuscitated *P. gingivalis* are shown compared to one another at four time points. Differences are analyzed using the two-way ANOVA with Tukey’s post-test data comparison.

We further investigated the culturability of intracellular *P. gingivalis* in infected HCAECs by lysing the cells at 6-, 24-, 48-, or 72-hour post-infection. Serial 10-fold dilutions of each sample were plated on BAPs and incubated anaerobically to enumerate viable colony forming units after 10 days of incubation. The lysates collected after 6 hours post-infection showed that HCAECs infected with control and resuscitated *P. gingivalis* cultures resulted in significantly higher CFU/mL than those of HCAECs infected with VBNC-state *P. gingivalis*. Significant differences between these groups were also observed in subsequent time points until the 72 hour time point was reached (Figs 4b, 5b, and 6b). By the 48-hour time point, a significant downward trend of recoverable colony forming units was observed in all three conditions compared to 6- and 24-hour time points.At the 72-hour time point, colony forming units were undetectable in all three groups (Fig 7). We demonstrated that *P. gingivalis* was incapable of growing under the atmospheric and nutritional conditions used in this host cell invasion assay (S6 Fig), but do not know if it was capable of replication once inside the host cell.

## Discussion

In recent years, diseases affecting distal organs have been associated with *P. gingivalis* infection [20]. This microorganism has been implicated in a range of conditions such as atherosclerosis, Alzheimer’s disease, rheumatoid arthritis, bacterial vaginosis, and various cancers [8,21]. The potential, wide-reaching impact of *P. gingivalis* on these various organs underscores its ability to exert pathogenic effects far from its primary habitat in the oral cavity [22]. Although various virulence factors have been studied extensively [23–26], the mechanism(s) by which *P. gingivalis* travels throughout the body and promotes disease in distant organs remains poorly understood. In this study, we demonstrated that *P. gingivalis* can enter a semi-dormant state, specifically, the VBNC state, which may enable it to survive under adverse conditions and evade host defenses during its systemic dissemination.

The VBNC state is a well-documented survival strategy in many bacteria [27], allowing them to remain minimally active metabolically and retain the potential for pathogenicity but lose the ability to form colonies under standard laboratory conditions, making them undetectable by standard laboratory methods. More than fifty diverse human pathogens have been reported to be able to enter the VBNC state, including major pathogenic species like *Escherichia coli*, *Vibrio cholerae*, *Helicobacter pylori* and *Listeria monocytogenes*, among many others [28,29]. The presence of VBNC bacteria poses significant public health risks because these dormant bacteria can resuscitate under suitable conditions, potentially regaining virulence and leading to disease relapse even in cases considered pathogen-free by culture-based diagnostics [30]. This state has not been fully studied in an oral anaerobic pathogen such as *P. gingivalis*, making our investigation a novel contribution to the understanding of *P. gingivalis’s* pathogenic capabilities.

We first investigated the ability of *P. gingivalis* to enter the VBNC state by exposing the bacterium to oxidative stress using H_2_O_2_, a common inducer of the VBNC state in other bacteria. The conditions we selected with regard to hydrogen peroxide concentration and period of exposure generally conform to conditions expected inside a phagolysosome [31]. As human monocyte-derived dendritic cells produce millimolar concentrations of reactive oxygen species (ROS) in phagosomes per second [31]. To confirm the VBNC state in *P. gingivalis*, we employed traditional culture methods and fluorescence-based microscopic techniques. While no or very few colonies were observed on BAPs from peroxide-treated samples, indicating a lack of culturability, the use of live/dead staining revealed the presence of viable bacteria. Confocal microscope images revealed that the hydrogen peroxide-treated cultures contained a high percentage of live *P. gingivalis* cells despite their inability to form colonies on BAPs (Fig 1, and Table 1). Further, it was shown that incubation of hydrogen peroxide-treated *P. gingivalis* with 1 mM pyruvate significantly increased the number of cultivable *P. gingivalis* recoverable, indicating of this compound could resuscitate VBNC *P. gingivalis* to a cultivable state. These findings were further supported by flow cytometry, which confirmed and quantified the percentage of live/dead *P. gingivalis* in each condition. The flow cytometry data demonstrated that, following H_2_O_2_ treatment, a considerable proportion of *P. gingivalis* cells (at least 62%) remained viable (Fig 2). The consistency of these results across repeated experiments suggests that the VBNC state in *P. gingivalis* is a stable and reproducible phenomenon induced by oxidative stress. Our findings align with previous reports on other bacterial species where oxidative stress has been shown to trigger the VBNC state as a protective response to hostile environments [32]. This stress-induced transition allows bacteria to persist in a dormant-like state until conditions become favorable for growth and reproduction.

The finding that hydrogen peroxide can induce a VBNC state in *P. gingivalis* suggests a possible mechanism by which immune cells such as macrophages and neutrophils may contribute to this microorganism’s persistence and spreading. During phagocytosis, these cells generate ROS, including hydrogen peroxide, to kill pathogens. However, *P. gingivalis* may evade destruction by quickly entering a VBNC state in response to oxidative stress. Once internalized, it is conceivable that VBNC *P. gingivalis* may be transported by immune cells to distal tissues, potentially contributing to systemic disease either upon resuscitation or while still in the VBNC state. It is also possible that VBNC *P. gingivalis* can survive and transit directly through the bloodstream.

An important aspect of our study was the ability to resuscitate VBNC-state *P. gingivalis* using sodium pyruvate, which may exert its effect by serving as an antioxidant and/or as a metabolic stimulant [33,34]. Sodium pyruvate has been demonstrated to promote the resuscitation of VBNC bacteria, particularly in species such as *Escherichia coli* and *Salmonella enteritidis*, by serving as both a carbon source and a crucial metabolic activator [35,36]. Treatment with 1 mM sodium pyruvate for 90 minutes successfully resuscitated VBNC-state *P. gingivalis* cells, as evidenced by the formation of colonies on BAPs (Fig 1a). This resuscitation process confirmed the viability of the VBNC *P. gingivalis* cells and demonstrated that the VBNC state in *P. gingivalis* is reversible. Based on this data, the efficiency of resuscitation appeared similar with *P. gingivalis* cells treated with pyruvate immediately after peroxide treatment (Fig 1) and *P. gingivalis* cells allowed 3 hours recovery in TSB before the addition of pyruvate (S1 Fig), suggesting that transition into the VBNC state is rapid for *P. gingivalis*. Additional studies to determine the kinetics of this transition will need to be performed.

The invasive potential of VBNC-state and resuscitated *P. gingivalis* relative to control cells was then assessed using a human coronary artery endothelial cell (HCAEC) invasion assay. The results showed that *P. gingivalis* in both VBNC and resuscitated states retained their ability to invade host cells, with invasion comparable to that of control *P. gingivalis* at all time points tested (Fig 3 and S2-S4 Fig). This finding has significant implications, as it suggests that *P. gingivalis* can remain infectious even in the VBNC state, posing a continued threat to the host. The ability of VBNC-state *P. gingivalis* cells to resuscitate and maintain their pathogenicity under favorable conditions is particularly concerning in the context of chronic and recurrent infections.

Using live/dead staining, we observed that the internalized *P. gingivalis* within HCAECs exhibited both live and dead forms over 72 hours, regardless of their initial state (Figs 4-6a). The significant changes observed in recoverable colony-forming units obtained from infected HCAEC lysates over time provided further insights into the physiological state of intracellular *P. gingivalis*. Bearing in mind that samples of VBNC *P. gingivalis* created by treatment with H_2_O_2_ produced virtually no colony forming units on BAPs, it was noteworthy that lysates of host cells infected with the same VBNC state *P. gingivalis* cells showed relatively large numbers of CFUs that were only one to two logs lower than host cells infected with control or resuscitated bacteria (Fig 7). This was the case at 6, 24, and 48 hours, although all three groups showed a downward trend in recoverable CFUs beginning with the 48 hour sample. By 72 hours, no cultivable *P. gingivalis* cells could be recoverable from any of the groups even though live/dead staining continued to show the presence of viable bacteria inside the host cells. These findings suggest that within the intracellular environment, some or all invading VBNC-state *P. gingivalis* may become resuscitated by 6 hours after host cell invasion and then enter a new VBNC state between 48 and 72 hours. Our findings align with previous studies [37] which showed that *P. gingivalis* can persist within host cells in a form detectable by immunostaining and microscopy yet remain unculturable using standard in vitro techniques. At the time that data was reported, however, the VBNC state had not been recognized for *P. gingivalis*. In later studies [11,38], we detected high levels of *P. gingivalis* genomic DNA in atherosclerotic plaques by Q-PCR, but no viable bacteria could be recovered on blood agar plates, which is also consistent with our observations in the present study.

## Conclusions

Our study demonstrated that *P. gingivalis* can enter a VBNC state when exposed to H₂O₂, an oxidative stressor. The study also showed that *P. gingivalis* also enters the VBNC state during infection of primary HCAEC cells. It is possible that oxidative stress and possibly other conditions present within the host cell were responsible for inducing VBNC formation inside HCAECs. Furthermore, the resuscitation of VBNC-state *P. gingivalis* back to a culturable state was achieved using sodium pyruvate, which may exert its effect as an antioxidant or a metabolic stimulant [33,34]. This finding is particularly significant as it underscores the reversible nature of the VBNC state, highlighting a potential pathway for bacterial recovery under favorable conditions. The pathogenic potential of *P. gingivalis* is intimately linked to its ability to invade host tissues. Importantly, our study also found that *P. gingivalis* in both VBNC and resuscitated states displayed invasion capabilities in a manner similar to control *P. gingivalis* cells.

Our observations underscore the adaptability of *P. gingivalis* and its ability to persist within host cells in a dormant-like state, suggesting the existence of a mechanism through which it can evade immune detection and potentially contribute to chronic infection. The persistence of *P. gingivalis* in a VBNC state within host tissues may contribute to the chronic nature of diseases such as periodontitis and atherosclerosis [39], where *P. gingivalis* is known to play a role. Moreover, the potential for resuscitation under favorable conditions, such as during periods of immunosuppression or other physiological changes, could lead to the recurrence of infection and disease progression. Interestingly, we also noted that the confocal images as seen in Fig 5a show the presence of aggregated *P. gingivalis* within the host cells, suggesting that these bacteria may be engaging in secondary interactions that may be the result of entering into the VBNC state. The formation of such aggregates may indicate the formation of biofilm-like structures or other protective mechanisms that also contribute to the ability of *P. gingivalis* to persist within host cells [40].

As this investigation is the first to report the ability of an obligate anaerobic pathogen to enter a VBNC state, we predict that other anaerobic species will be found to be capable of a VBNC lifestyle.

Our on-going and future research will focus on further elucidating the molecular mechanisms underlying the VBNC state in *P. gingivalis,* determining the significance of the VBNC state for extraoral dissemination, and exploring potential therapeutic strategies to target VBNC-state bacteria at the molecular level. The development of treatments that can either prevent the transition into the VBNC state or the ability to resuscitate from it could provide rational approaches to eradication of VBNC bacteria and have profound implications for the management of chronic infections and systemic diseases associated with *P. gingivalis*.

## Supporting information

S1 Fig Calibration for the flow cytometry method of counting live and dead bacteria. Live is green, Dead is purple. The flow cytometry measure included height, area, and stained fluorescence to show the count results: a) *P. gingivalis* no stain control (NC), b) H_2_O_2_-treated *P. gingivalis* no stain control (H_2_O_2_ UN), and c) H_2_O_2_-treated and sodium pyruvate-treated *P. gingivalis* no stain control (Pyruvate UN).
(TIF)

S2 Fig Invasion of HCAECs at 24 hours post-infection by *P. gingivalis* in three different physiological states. Top: Mono images of DAPI staining of A) Uninfected HCAEC control; B) HCAEC cells infected with control *P. gingivalis*; C) HCAEC cells infected with VBNC-state *P. gingivalis*; and D) HCAEC cells infected with pyruvate resuscitated *P. gingivalis*. Bottom: Merged color images of DAPI staining (blue) with MitoTracker Red CMXRos (red) to highlight HCAEC boundary.
(TIF)

S3 Fig Invasion of HCAECs at 48 hours post-infection by *P. gingivalis* in three different physiological states. Top: Mono images of DAPI staining of A) Uninfected HCAEC control; B) HCAEC cells infected with control *P. gingivalis*; C) HCAEC cells infected with VBNC-state *P. gingivalis*; and D) HCAEC cells infected with pyruvate resuscitated *P. gingivalis*. Bottom: Merged color images of DAPI staining (blue) with MitoTracker Red CMXRos (red) to highlight HCAEC boundary.
(TIF)

S4 Fig Invasion of HCAECs at 72 hours post-infection by *P. gingivalis* in three different physiological states. Top: Mono images of DAPI staining of A) Uninfected HCAEC control; B) HCAEC cells infected with control *P. gingivalis*; C) HCAEC cells infected with VBNC-state *P. gingivalis*; and D) HCAEC cells infected with pyruvate resuscitated *P. gingivalis*. Bottom: Merged color images of DAPI staining (blue) with MitoTracker Red CMXRos (red) to highlight HCAEC boundary.
(TIF)

S5 Fig Micrographs of uninfected control HCAEC. Live/dead staining of uninfected HCAEC at 6, 24, 48, and 72 hours; i) SYTO 9 (green), ii) PI (red). Scale bar = 10μm.
(TIF)

S6 Fig Aerobic and Anaerobic incubation of *P. gingivalis* in TSB and HCAEC media EGM. OD_550_ of *P. gingivalis* after incubating aerobically in TSB or EGM-2 MV, in aerobic and anaerobic conditions in a time point measurement. The data shown are the means and standard deviations of three independent samples.
(TIF)

S1 Data Pg Flow Cytometry Data and Protocol. Detailed procedural protocol for flow cytomery.
(PDF)

S2 Data Pg Manuscript Raw CFU Data. Unprocessed colony counts for experimental replicates.
(XLSX)

## References

[1] Liu J, Yang L, Kjellerup BV, Xu Z. Viable but nonculturable (VBNC) state, an underestimated and controversial microbial survival strategy. Trends Microbiol. 2023;31(10):1013–23. doi: 10.1016/j.tim.2023.04.009 37225640

[2] Oliver JD. The public health significance of viable but nonculturable bacteria. Nonculturable microorganisms in the environment. 2000. p. 277–300.

[3] Xu HS, Roberts N, Singleton FL, Attwell RW, Grimes DJ, Colwell RR. Survival and viability of nonculturableEscherichia coli andVibrio cholerae in the estuarine and marine environment. Microb Ecol. 1982;8(4):313–23. doi: 10.1007/BF02010671 24226049

[4] Dong K, Pan H, Yang D, Rao L, Zhao L, Wang Y, et al. Induction, detection, formation, and resuscitation of viable but non-culturable state microorganisms. Compr Rev Food Sci Food Saf. 2020;19(1):149–83. doi: 10.1111/1541-4337.12513 33319518

[5] Progulske-Fox A, Chukkapalli SS, Getachew H, Dunn WA, Oliver JD. VBNC, previously unrecognized in the life cycle of Porphyromonas gingivalis? J Oral Microbiol. 2022;14(1):1952838. doi: 10.1080/20002297.2021.1952838 35035782 PMC8759725

[6] Yang D, Wang Y, Zhao L, Rao L, Liao X. Extracellular pH decline introduced by high pressure carbon dioxide is a main factor inducing bacteria to enter viable but non-culturable state. Food Res Int. 2022;151:110895. doi: 10.1016/j.foodres.2021.110895 34980417

[7] Bagatella S, Monney C, Gross N, Bernier Gosselin V, Schüpbach-Regula G, Hemphill A, et al. Intravacuolar persistence in neutrophils facilitates Listeria monocytogenes spread to co-cultured cells. mBio. 2025;16(4):e0270024. doi: 10.1128/mbio.02700-24 40067021 PMC11980584

[8] Bahar B, Kanagasingam S, Tambuwala MM, Aljabali AAA, Dillon SA, Doaei S, et al. Porphyromonas gingivalis (W83) Infection Induces Alzheimer’s Disease-Like Pathophysiology in Obese and Diabetic Mice. J Alzheimers Dis. 2021;82(3):1259–75. doi: 10.3233/JAD-210465 34151813

[9] Carter CJ, France J, Crean S, Singhrao SK. The Porphyromonas gingivalis/Host Interactome Shows Enrichment in GWASdb Genes Related to Alzheimer’s Disease, Diabetes and Cardiovascular Diseases. Front Aging Neurosci. 2017;9:408. doi: 10.3389/fnagi.2017.00408 29311898 PMC5732932

[10] Lamont RJ, Fitzsimonds ZR, Wang H, Gao S. Role of Porphyromonas gingivalis in oral and orodigestive squamous cell carcinoma. Periodontol 2000. 2022;89(1):154–65. doi: 10.1111/prd.12425 35244980 PMC9439709

[11] Kozarov EV, Dorn BR, Shelburne CE, Dunn WA Jr, Progulske-Fox A. Human atherosclerotic plaque contains viable invasive Actinobacillus actinomycetemcomitans and Porphyromonas gingivalis. Arterioscler Thromb Vasc Biol. 2005;25(3):e17-8. doi: 10.1161/01.ATV.0000155018.67835.1a 15662025

[12] Helaine S, Kugelberg E. Bacterial persisters: formation, eradication, and experimental systems. Trends Microbiol. 2014;22(7):417–24. doi: 10.1016/j.tim.2014.03.008 24768561

[13] Kumbar VM, Peram MR, Kugaji MS, Shah T, Patil SP, Muddapur UM, et al. Effect of curcumin on growth, biofilm formation and virulence factor gene expression of Porphyromonas gingivalis. Odontology. 2021;109(1):18–28. doi: 10.1007/s10266-020-00514-y 32279229

[14] Ciaston I, Budziaszek J, Satala D, Potempa B, Fuchs A, Rapala-Kozik M, et al. Proteolytic Activity-Independent Activation of the Immune Response by Gingipains from Porphyromonas gingivalis. mBio. 2022;13(3):e0378721. doi: 10.1128/mbio.03787-21 35491845 PMC9239244

[15] Zhang Z, Liu D, Liu S, Zhang S, Pan Y. The Role of Porphyromonas gingivalis Outer Membrane Vesicles in Periodontal Disease and Related Systemic Diseases. Front Cell Infect Microbiol. 2021;10:585917. doi: 10.3389/fcimb.2020.585917 33585266 PMC7877337

[16] Bertelsen CV, Franco JC, Skands GE, Dimaki M, Svendsen WE. Investigating the Use of Impedance Flow Cytometry for Classifying the Viability State of E. coli. Sensors (Basel). 2020;20(21):6339. doi: 10.3390/s20216339 33172055 PMC7664255

[17] Davey H, Guyot S. Estimation of Microbial Viability Using Flow Cytometry. Curr Protoc Cytom. 2020;93(1):e72. doi: 10.1002/cpcy.72 32289207

[18] Getachew H. The intracellular survival of Porphyromonas gingivalis in human coronary artery endothelial and smooth muscle cells. University of Florida; 2019.

[19] Malik A, Oludiran A, Poudel A, Alvarez OB, Woodward C, Purcell EB. RelQ-mediated alarmone signalling regulates growth, stress-induced biofilm formation and spore accumulation in Clostridioides difficile. Microbiology. 2024;170(7):001479. 39028551 10.1099/mic.0.001479 PMC11317968

[20] Beydoun MA, Beydoun HA, Hossain S, El-Hajj ZW, Weiss J, Zonderman AB. Clinical and Bacterial Markers of Periodontitis and Their Association with Incident All-Cause and Alzheimer’s Disease Dementia in a Large National Survey. J Alzheimers Dis. 2020;75(1):157–72. doi: 10.3233/JAD-200064 32280099 PMC11008556

[21] Díaz-Zúñiga J, More J, Melgar-Rodríguez S, Jiménez-Unión M, Villalobos-Orchard F, Muñoz-Manríquez C, et al. Alzheimer’s Disease-Like Pathology Triggered by Porphyromonas gingivalis in Wild Type Rats Is Serotype Dependent. Front Immunol. 2020;11:588036. doi: 10.3389/fimmu.2020.588036 33240277 PMC7680957

[22] Lafuente Ibáñez de Mendoza I, Maritxalar Mendia X, Garcia de la Fuente AM, Quindos Andres G, Aguirre Urizar JM. Role of Porphyromonas gingivalis in oral squamous cell carcinoma development: A systematic review. J Periodont Res. 2020;55(1):13–22. 10.1111/jre.12691 31529626

[23] Derke RM, Barron AJ, Billiot CE, Chaple IF, Lapi SE, Broderick NA, et al. The Cu(II) Reductase RclA Protects Escherichia coli against the Combination of Hypochlorous Acid and Intracellular Copper. mBio. 2020;11(5):e01905-20. doi: 10.1128/mBio.01905-20 32994322 PMC7527725

[24] Buvelot H, Roth M, Jaquet V, Lozkhin A, Renzoni A, Bonetti E-J, et al. Hydrogen Peroxide Affects Growth of S. aureus Through Downregulation of Genes Involved in Pyrimidine Biosynthesis. Front Immunol. 2021;12:673985. doi: 10.3389/fimmu.2021.673985 34557184 PMC8454235

[25] da Cruz Nizer WS, Inkovskiy V, Versey Z, Strempel N, Cassol E, Overhage J. Oxidative Stress Response in Pseudomonas aeruginosa. Pathogens. 2021;10(9):1187. doi: 10.3390/pathogens10091187 34578219 PMC8466533

[26] Ghods S, Moradali MF, Duryea D, Walker AR, Davey ME. Growth of Porphyromonas gingivalis on human serum albumin triggers programmed cell death. J Oral Microbiol. 2022;15(1):2161182. doi: 10.1080/20002297.2022.2161182 36570975 PMC9788703

[27] Li L, Mendis N, Trigui H, Oliver JD, Faucher SP. The importance of the viable but non-culturable state in human bacterial pathogens. Front Microbiol. 2014;5:258. doi: 10.3389/fmicb.2014.00258 24917854 PMC4040921

[28] Xu HS, Roberts N, Singleton FL, Attwell RW, Grimes DJ, Colwell RR. Survival and viability of nonculturableEscherichia coli andVibrio cholerae in the estuarine and marine environment. Microb Ecol. 1982;8(4):313–23. doi: 10.1007/BF02010671 24226049

[29] Adams BL, Bates TC, Oliver JD. Survival of Helicobacter pylori in a natural freshwater environment. Appl Environ Microbiol. 2003;69(12):7462–6. doi: 10.1128/AEM.69.12.7462-7466.2003 14660399 PMC310012

[30] İzgördü ÖK, Darcan C, Kariptaş E. Overview of VBNC, a survival strategy for microorganisms. 3 Biotech. 2022;12(11):307. doi: 10.1007/s13205-022-03371-4 36276476 PMC9526772

[31] Paardekooper LM, Dingjan I, Linders PTA, Staal AHJ, Cristescu SM, Verberk WCEP, et al. Human Monocyte-Derived Dendritic Cells Produce Millimolar Concentrations of ROS in Phagosomes Per Second. Front Immunol. 2019;10:1216. doi: 10.3389/fimmu.2019.01216 31191556 PMC6548834

[32] Prosdocimi EM, Arioli S, Mapelli F, Zeaiter Z, Fusi M, Daffonchio D, et al. Cell phenotype changes and oxidative stress response in Vibrio spp. induced into viable but non-culturable (VBNC) state. Ann Microbiol. 2023;73(1). doi: 10.1186/s13213-022-01703-6

[33] Moradali MF, Davey ME. Metabolic plasticity enables lifestyle transitions of Porphyromonas gingivalis. NPJ Biofilms Microbiomes. 2021;7(1):46. doi: 10.1038/s41522-021-00217-4 34031416 PMC8144566

[34] Paulini S, Fabiani FD, Weiss AS, Moldoveanu AL, Helaine S, Stecher B, et al. The Biological Significance of Pyruvate Sensing and Uptake in Salmonella enterica Serovar Typhimurium. Microorganisms. 2022;10(9):1751. doi: 10.3390/microorganisms10091751 36144354 PMC9504724

[35] Morishige Y, Fujimori K, Amano F. Differential resuscitative effect of pyruvate and its analogues on VBNC (viable but non-culturable) Salmonella. Microbes Environ. 2013;28(2):180–6. doi: 10.1264/jsme2.me12174 23595023 PMC4070669

[36] Vilhena C, Kaganovitch E, Grünberger A, Motz M, Forné I, Kohlheyer D, et al. Importance of Pyruvate Sensing and Transport for the Resuscitation of Viable but Nonculturable Escherichia coli K-12. J Bacteriol. 2019;201(3):e00610-18. doi: 10.1128/JB.00610-18 30420452 PMC6349089

[37] Li L, Michel R, Cohen J, Decarlo A, Kozarov E. Intracellular survival and vascular cell-to-cell transmission of Porphyromonas gingivalis. BMC Microbiol. 2008;8:26. doi: 10.1186/1471-2180-8-26 18254977 PMC2259307

[38] Kozarov E, Sweier D, Shelburne C, Progulske-Fox A, Lopatin D. Detection of bacterial DNA in atheromatous plaques by quantitative PCR. Microbes Infect. 2006;8(3):687–93. doi: 10.1016/j.micinf.2005.09.004 16513386

[39] Contaldo M, Itro A, Lajolo C, Gioco G, Inchingolo F, Serpico R. Overview on Osteoporosis, Periodontitis and Oral Dysbiosis: The Emerging Role of Oral Microbiota. Appl Sci. 2020;10(17):6000. doi: 10.3390/app10176000

[40] Chopra A, Bhat SG, Sivaraman K. Porphyromonas gingivalis adopts intricate and unique molecular mechanisms to survive and persist within the host: a critical update. J Oral Microbiol. 2020;12(1):1801090. doi: 10.1080/20002297.2020.1801090 32944155 PMC7482874

