# Supplementary figures and images for: Investigation of a viable but non-culturable state in Porphyromonas gingivalis and host cell invasion
Source: PLoS One. 2026 Jan 16;21(1):e0340605. doi: 10.1371/journal.pone.0340605 (PMC12810784; doi:10.1371/journal.pone.0340605)

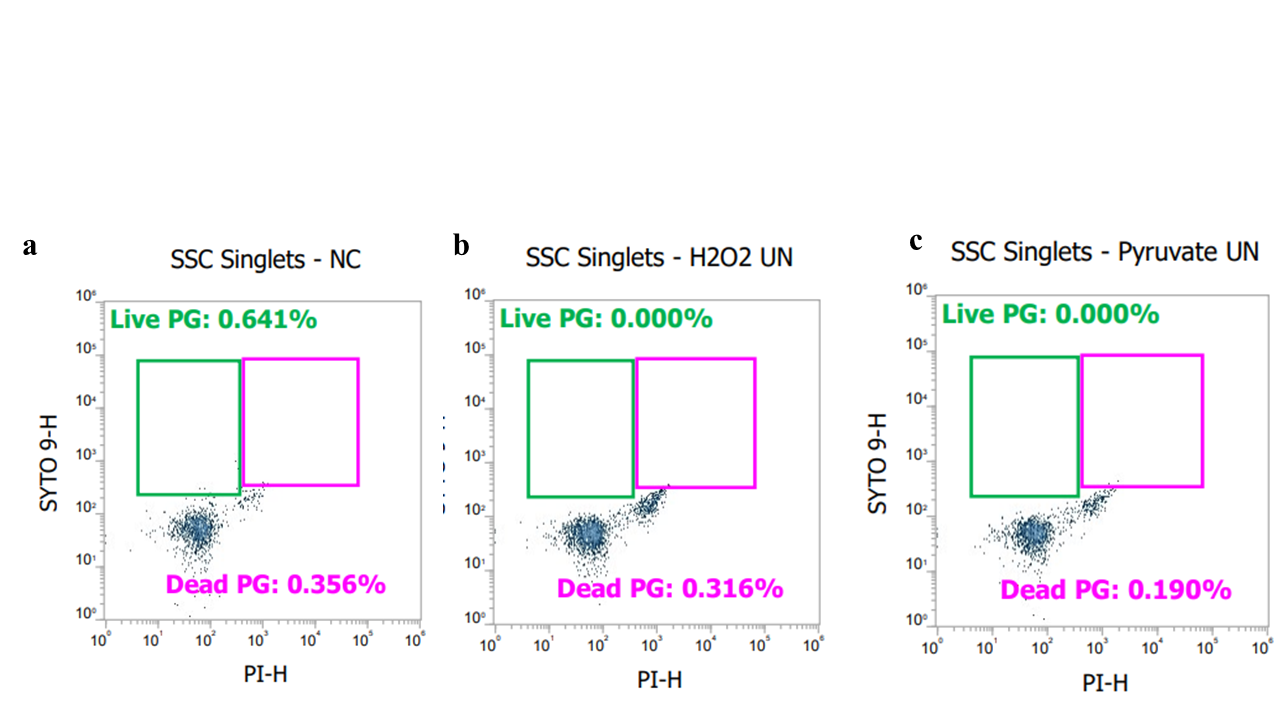

Supplement: S1 Fig — Live is green, Dead is purple. The flow cytometry measure included height, area, and stained fluorescence to show the count results: a) P. gingivalis no stain control (NC), b) H2O2-treated P. gingivalis no stain control (H2O2 UN), and c) H2O2-treated and sodium pyruvate-treated P. gingivalis no stain control (Pyruvate UN). (TIF) [file pone.0340605.s001.TIF]

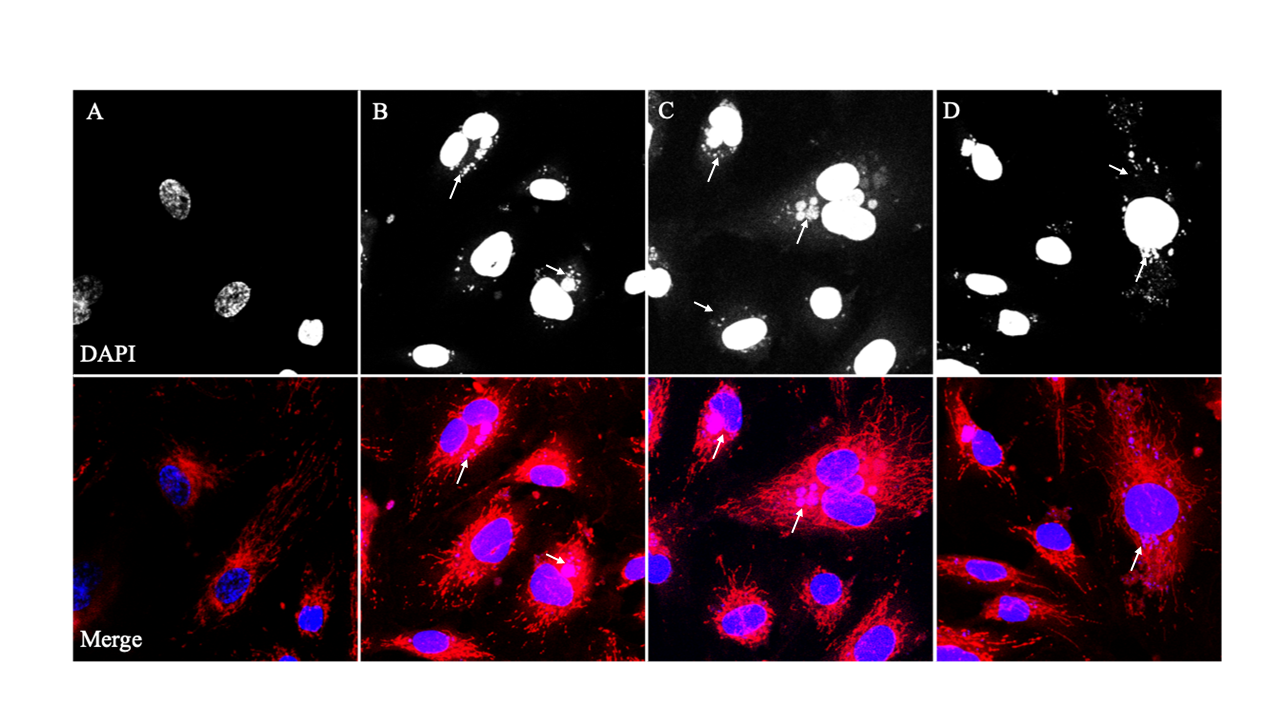

Supplement: S2 Fig — Top: Mono images of DAPI staining of A) Uninfected HCAEC control; B) HCAEC cells infected with control P. gingivalis; C) HCAEC cells infected with VBNC-state P. gingivalis; and D) HCAEC cells infected with pyruvate resuscitated P. gingivalis. Bottom: Merged color images of DAPI staining (blue) with MitoTracker Red CMXRos (red) to highlight HCAEC boundary. (TIF) [file pone.0340605.s002.TIF]

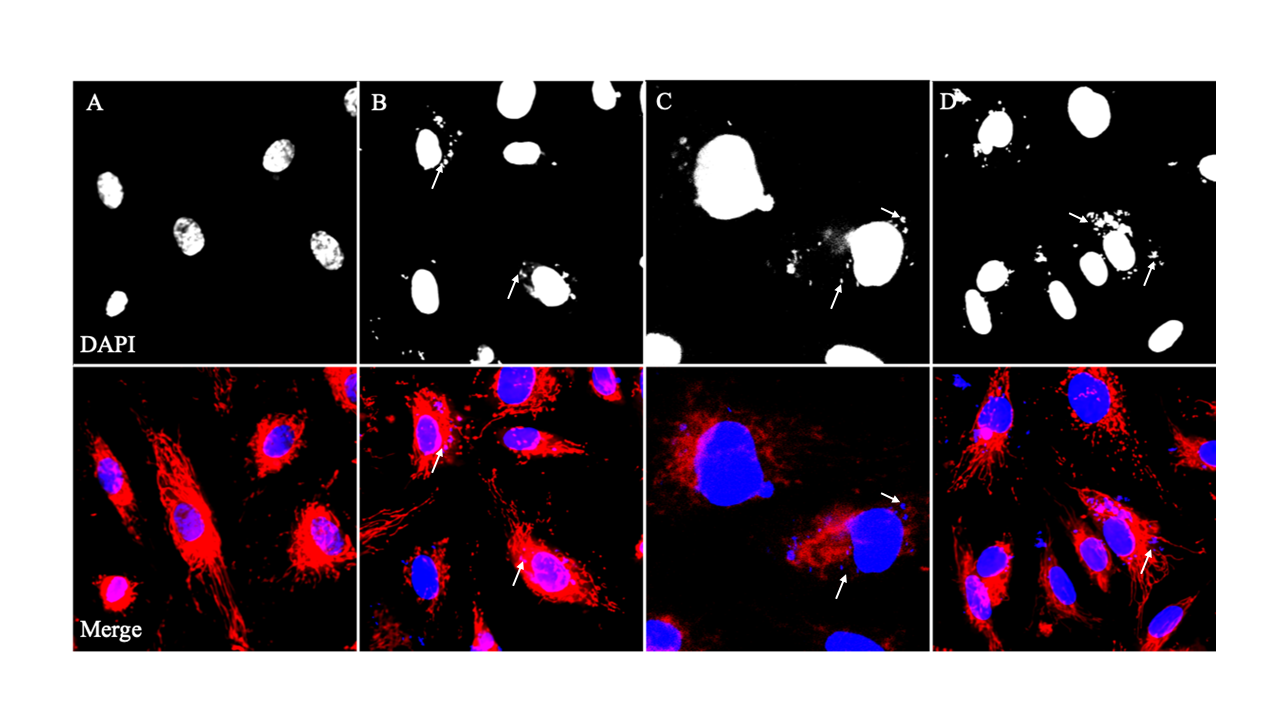

Supplement: S3 Fig — Top: Mono images of DAPI staining of A) Uninfected HCAEC control; B) HCAEC cells infected with control P. gingivalis; C) HCAEC cells infected with VBNC-state P. gingivalis; and D) HCAEC cells infected with pyruvate resuscitated P. gingivalis. Bottom: Merged color images of DAPI staining (blue) with MitoTracker Red CMXRos (red) to highlight HCAEC boundary. (TIF) [file pone.0340605.s003.TIF]

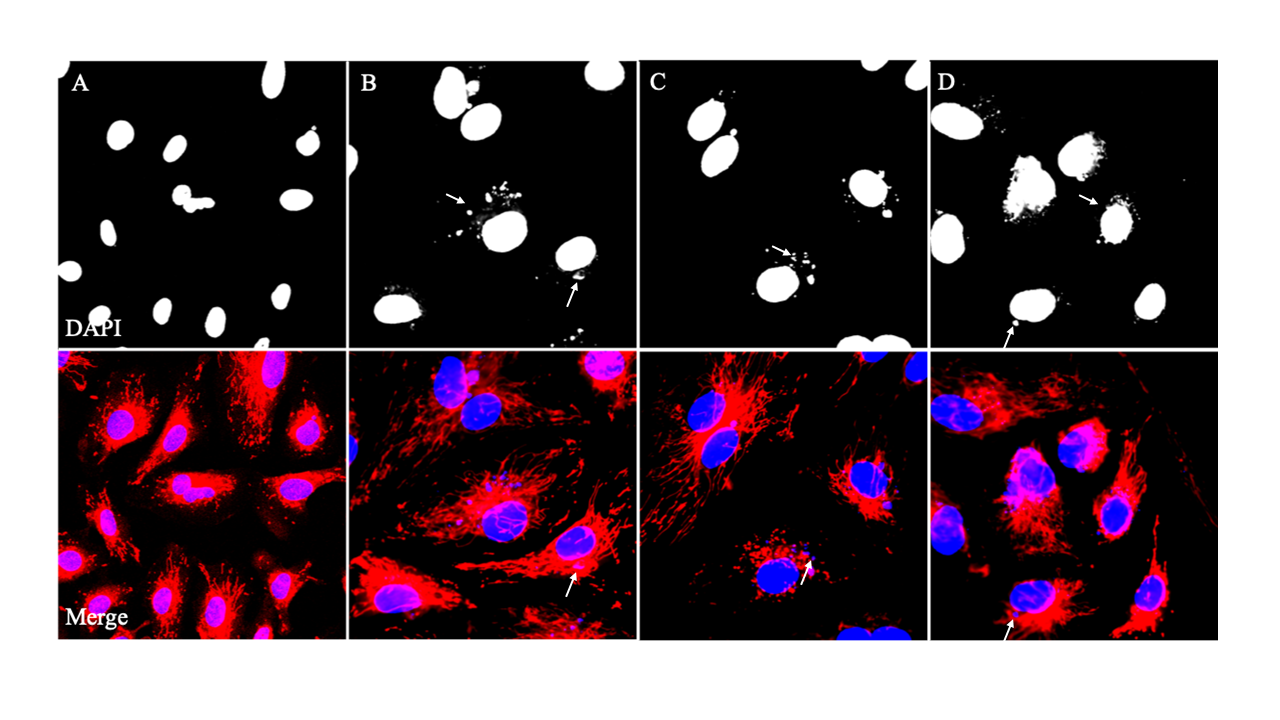

Supplement: S4 Fig — Top: Mono images of DAPI staining of A) Uninfected HCAEC control; B) HCAEC cells infected with control P. gingivalis; C) HCAEC cells infected with VBNC-state P. gingivalis; and D) HCAEC cells infected with pyruvate resuscitated P. gingivalis. Bottom: Merged color images of DAPI staining (blue) with MitoTracker Red CMXRos (red) to highlight HCAEC boundary. (TIF) [file pone.0340605.s004.TIF]

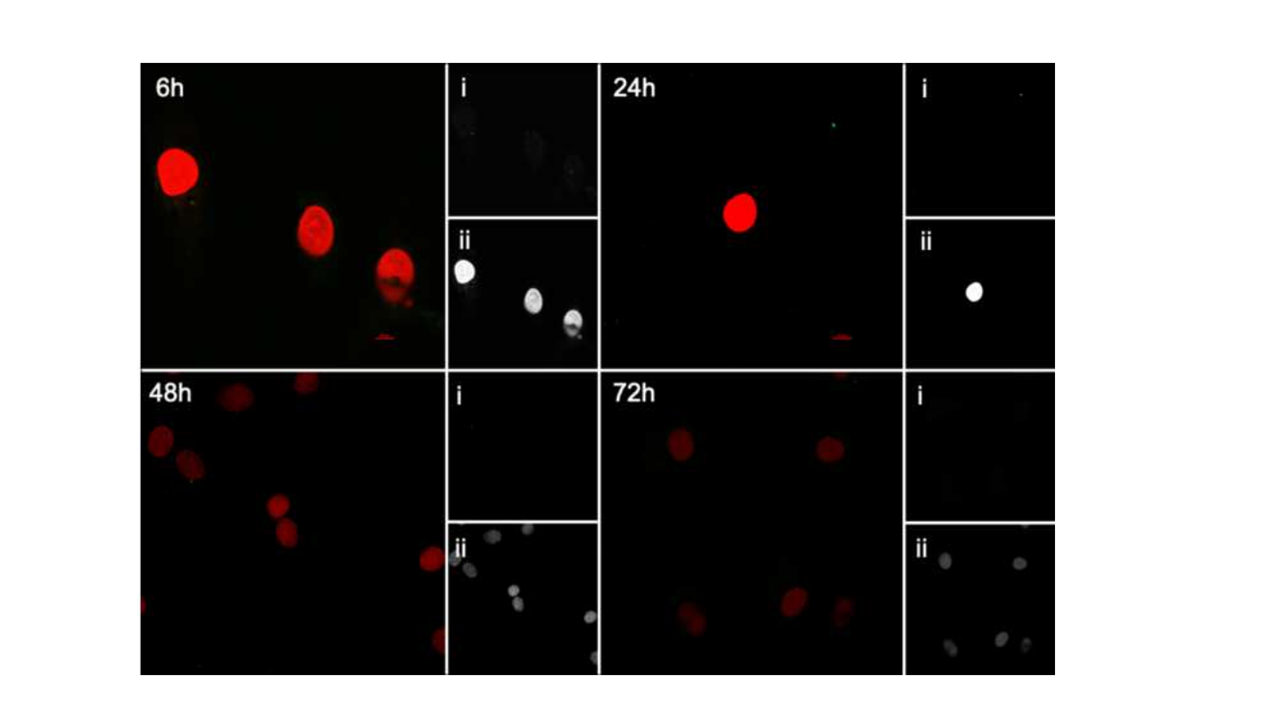

Supplement: S5 Fig — Live/dead staining of uninfected HCAEC at 6, 24, 48, and 72 hours; i) SYTO 9 (green), ii) PI (red). Scale bar = 10μm. (TIF) [file pone.0340605.s005.TIF]

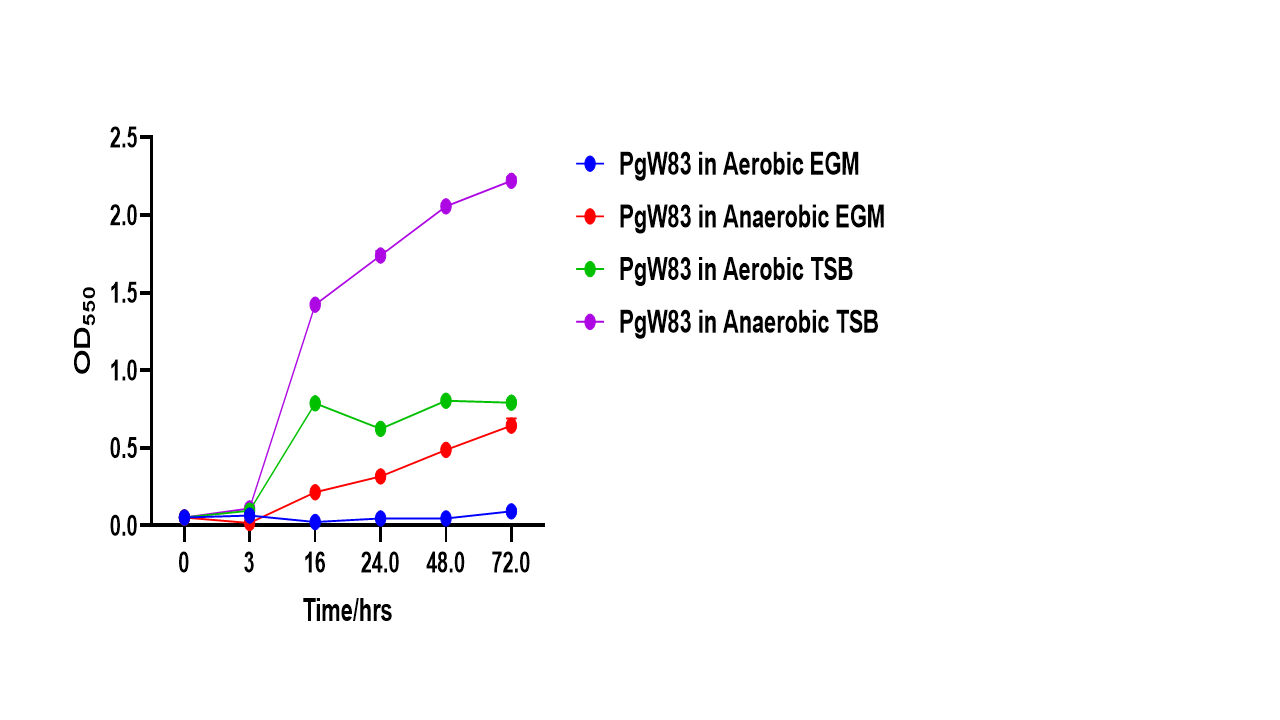

Supplement: S6 Fig — OD550 of P. gingivalis after incubating aerobically in TSB or EGM-2 MV, in aerobic and anaerobic conditions in a time point measurement. The data shown are the means and standard deviations of three independent samples. (TIF) [file pone.0340605.s006.TIF]
