# Supplementary material for: Investigation of a viable but non-culturable state in Porphyromonas gingivalis and host cell invasion
Source: PLoS One. 2026 Jan 16;21(1):e0340605. doi: 10.1371/journal.pone.0340605 (PMC12810784; doi:10.1371/journal.pone.0340605)
Supplement: S1 Data — Detailed procedural protocol for flow cytomery. (PDF) [file pone.0340605.s007.pdf]

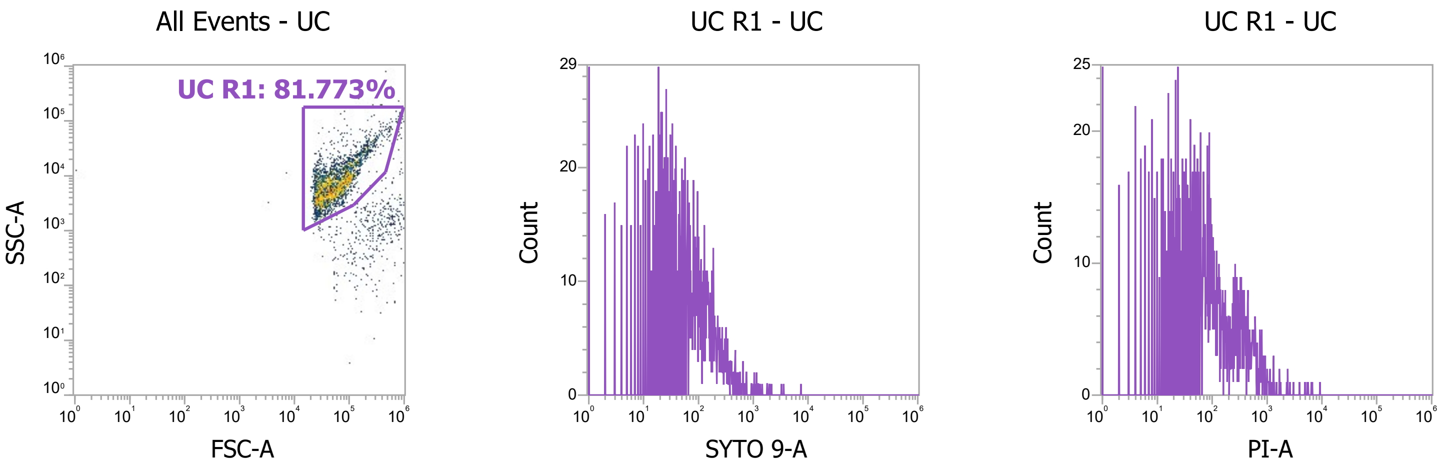
All Events - UC
UC R1: 81.773%
SSC-A
FSC-A
UC R1 - UC
Count
SYTO 9-A
UC R1 - UC
Count
PI-A

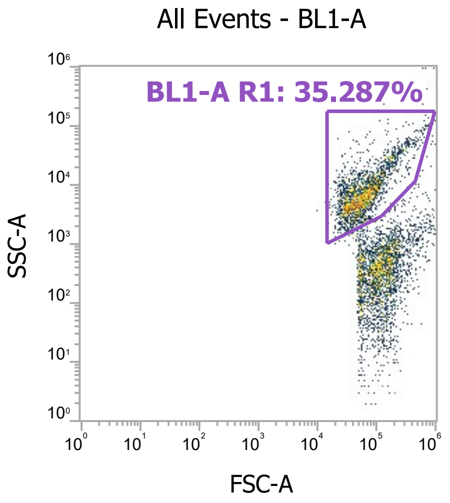

All Events - BL1-A
BL1-A R1: 35.287%
SSC-A
FSC-A

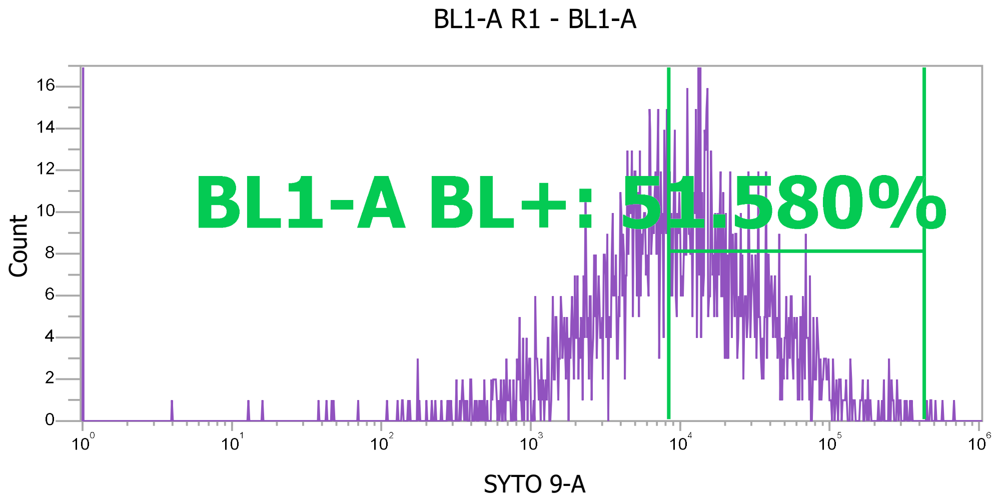

BL1-A R1 - BL1-A
BL1-A BL+: 51.580%
Count
SYTO 9-A

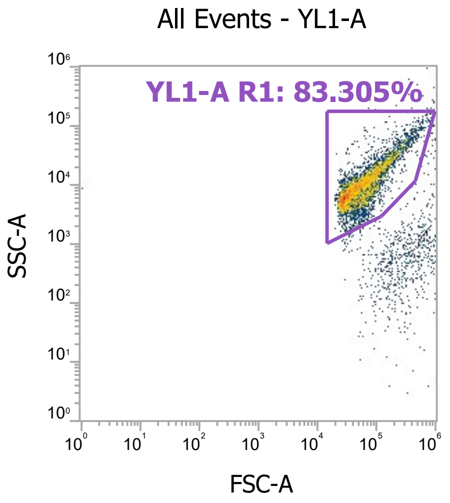
All Events - YL1-A
YL1-A R1: 83.305%
SSC-A
FSC-A

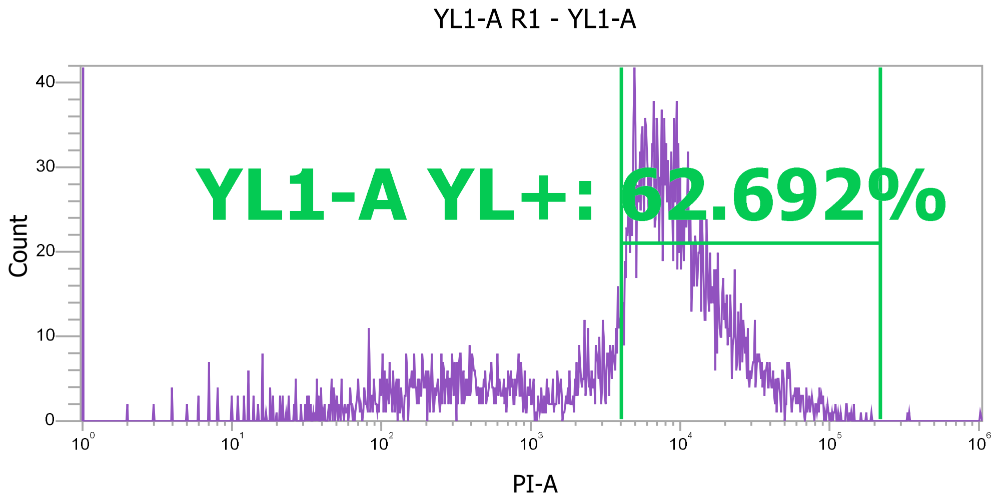
YL1-A R1 - YL1-A
YL1-A YL+: 62.692%
Count
PI-A

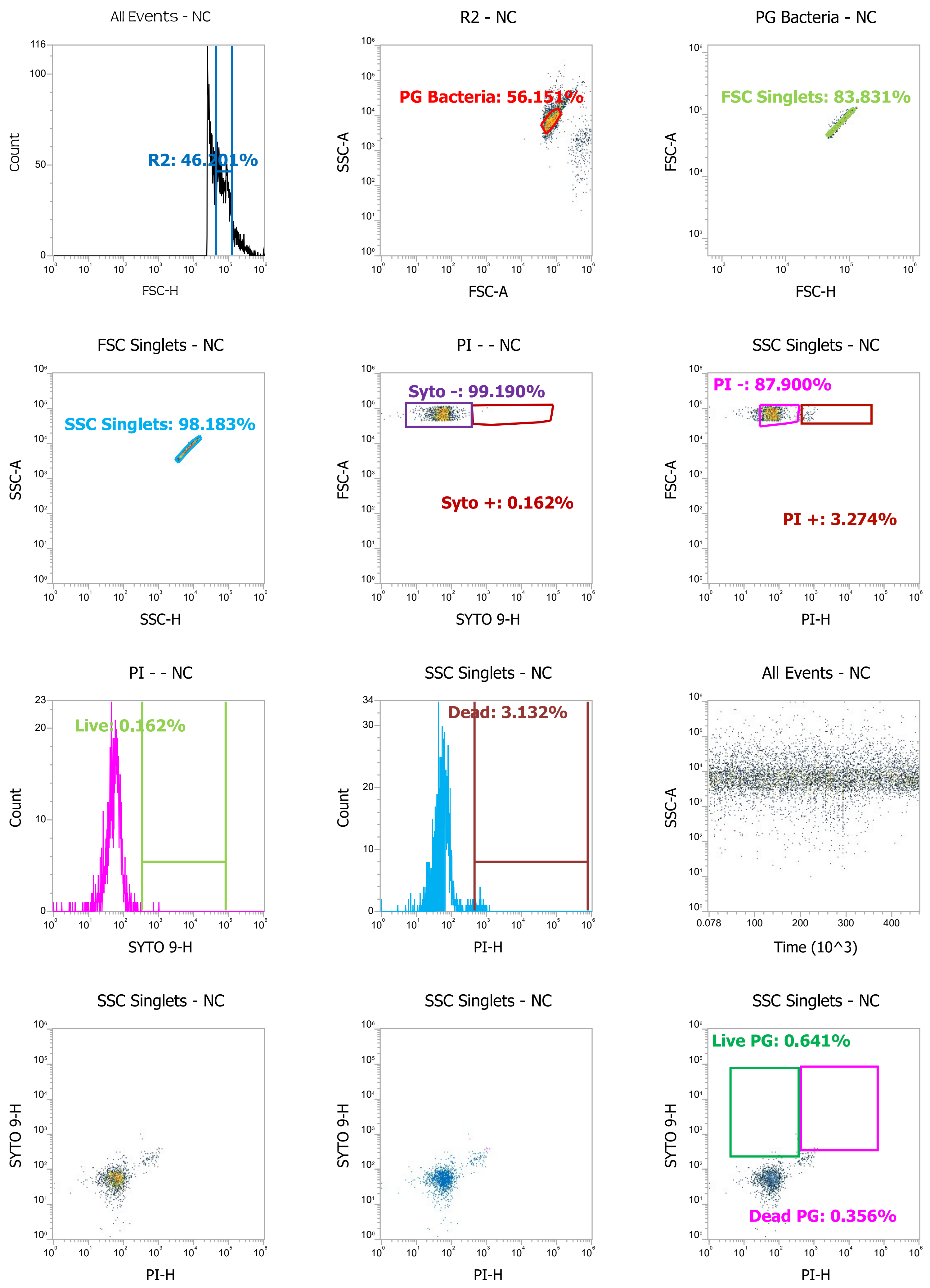

All Events - NC
R2: 46.201%
Count
FSC-H
R2 - NC
PG Bacteria: 56.151%
SSC-A
FSC-A
PG Bacteria - NC
FSC Singlets: 83.831%
FSC-A
FSC-H
FSC Singlets - NC
SSC Singlets: 98.183%
SSC-A
SSC-H
PI - - NC
Syto -: 99.190%
Syto +: 0.162%
FSC-A
SYTO 9-H
SSC Singlets - NC
PI -: 87.900%
PI +: 3.274%
FSC-A
PI-H
PI - - NC
Live: 0.162%
Count
SYTO 9-H
SSC Singlets - NC
Dead: 3.132%
Count
PI-H
All Events - NC
SSC-A
Time (10^3)
SSC Singlets - NC
SYTO 9-H
PI-H
SSC Singlets - NC
SYTO 9-H
PI-H
SSC Singlets - NC
Live PG: 0.641%
Dead PG: 0.356%
SYTO 9-H
PI-H

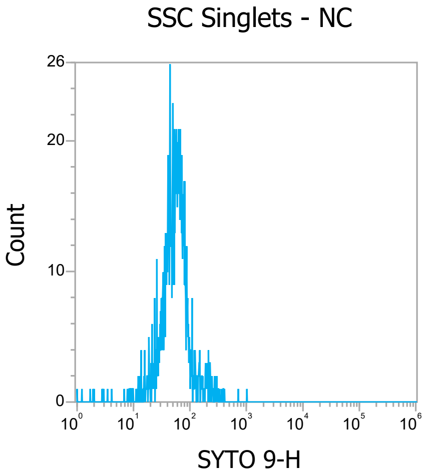

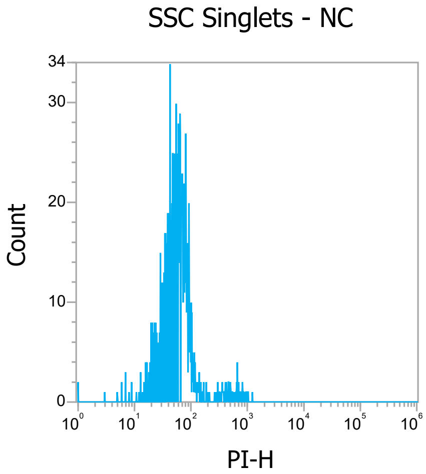

Experiment: **1st VBNC FC 4.2.24**
Group: **Group**
Sample: **NC**
Time Recorded: **12:24:32**

Name	Gate	Autogate Status	X Parameter	Y Parameter	Count	%Total	%Gated
All Events	All Events	N/A	N/A	N/A	6,580	100.000	100.000
R2	R2	N/A	FSC - FSC-H		3,040	46.201	46.201
PG Bacteria	PG Bacteria	N/A	FSC - FSC-A	SSC - SSC-A	1,707	25.942	56.151
FSC Singlets	FSC Singlets	N/A	FSC - FSC-H	FSC - FSC-A	1,431	21.748	83.831
SSC Singlets	SSC Singlets	N/A	SSC - SSC-H	SSC - SSC-A	1,405	21.353	98.183
PI -	PI -	N/A	PI - YL1-H	FSC - FSC-A	1,235	18.769	87.900
Syto +	Syto +	N/A	SYTO 9 - BL1-H	FSC - FSC-A	2	0.030	0.162
Syto -	Syto -	N/A	SYTO 9 - BL1-H	FSC - FSC-A	1,225	18.617	99.190
Live	Live	N/A	SYTO 9 - BL1-H		2	0.030	0.162
PI +	PI +	N/A	PI - YL1-H	FSC - FSC-A	46	0.699	3.274
Dead	Dead	N/A	PI - YL1-H		44	0.669	3.132
Live PG	Live PG	N/A	PI - YL1-H	SYTO 9 - BL1-H	9	0.137	0.641
Dead PG	Dead PG	N/A	PI - YL1-H	SYTO 9 - BL1-H	5	0.076	0.356

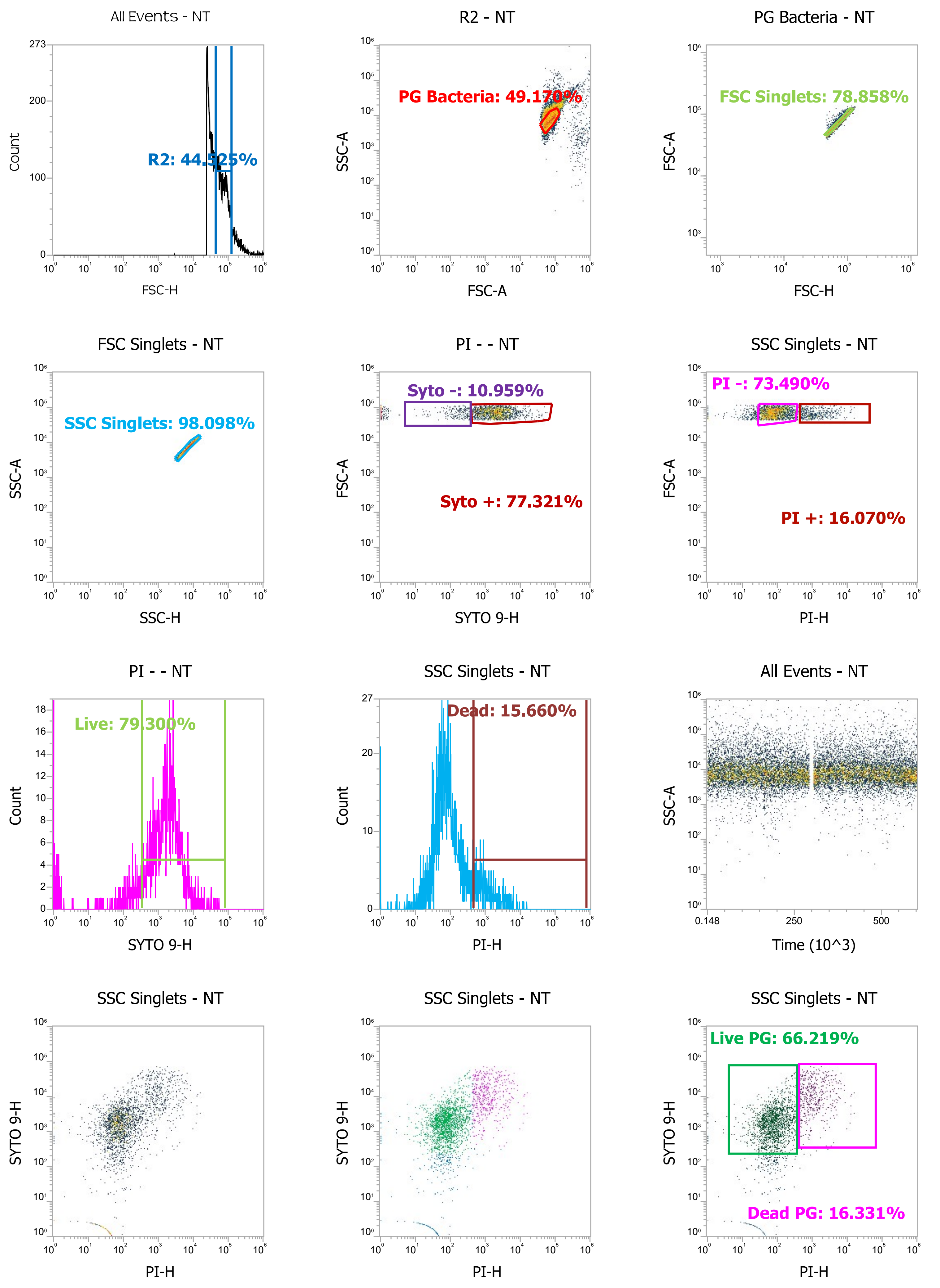

All Events - NT
R2: 44.525%
Count
FSC-H
R2 - NT
PG Bacteria: 49.170%
SSC-A
FSC-A
PG Bacteria - NT
FSC Singlets: 78.858%
FSC-A
FSC-H
FSC Singlets - NT
SSC Singlets: 98.098%
SSC-A
SSC-H
PI - - NT
Syto -: 10.959%
Syto +: 77.321%
FSC-A
SYTO 9-H
SSC Singlets - NT
PI -: 73.490%
PI +: 16.070%
FSC-A
PI-H
PI - - NT
Live: 79.300%
Count
SYTO 9-H
SSC Singlets - NT
Dead: 15.660%
Count
PI-H
All Events - NT
SSC-A
Time (10^3)
SSC Singlets - NT
SYTO 9-H
PI-H
SSC Singlets - NT
SYTO 9-H
PI-H
SSC Singlets - NT
Live PG: 66.219%
Dead PG: 16.331%
SYTO 9-H
PI-H

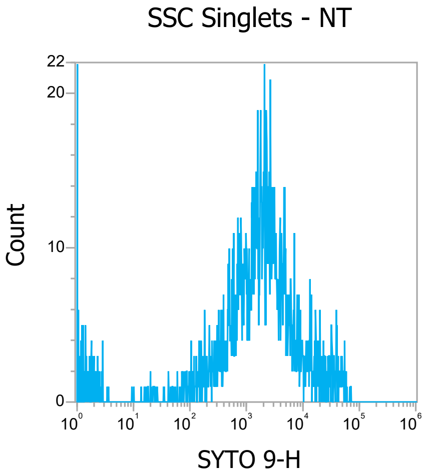

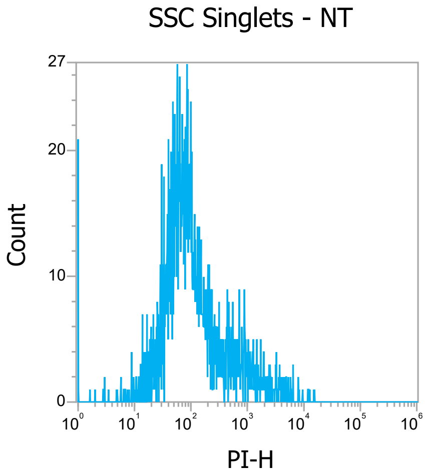

Experiment: **1st VBNC FC 4.2.24**
Group: **Group**
Sample: **NT**
Time Recorded: **12:41:29**

Name	Gate	Autogate Status	X Parameter	Y Parameter	Count	%Total	%Gated
All Events	All Events	N/A	N/A	N/A	15,836	100.000	100.000
R2	R2	N/A	FSC - FSC-H		7,051	44.525	44.525
PG Bacteria	PG Bacteria	N/A	FSC - FSC-A	SSC - SSC-A	3,467	21.893	49.170
FSC Singlets	FSC Singlets	N/A	FSC - FSC-H	FSC - FSC-A	2,734	17.264	78.858
SSC Singlets	SSC Singlets	N/A	SSC - SSC-H	SSC - SSC-A	2,682	16.936	98.098
PI -	PI -	N/A	PI - YL1-H	FSC - FSC-A	1,971	12.446	73.490
Syto +	Syto +	N/A	SYTO 9 - BL1-H	FSC - FSC-A	1,524	9.624	77.321
Syto -	Syto -	N/A	SYTO 9 - BL1-H	FSC - FSC-A	216	1.364	10.959
Live	Live	N/A	SYTO 9 - BL1-H		1,563	9.870	79.300
PI +	PI +	N/A	PI - YL1-H	FSC - FSC-A	431	2.722	16.070
Dead	Dead	N/A	PI - YL1-H		420	2.652	15.660
Live PG	Live PG	N/A	PI - YL1-H	SYTO 9 - BL1-H	1,776	11.215	66.219
Dead PG	Dead PG	N/A	PI - YL1-H	SYTO 9 - BL1-H	438	2.766	16.331

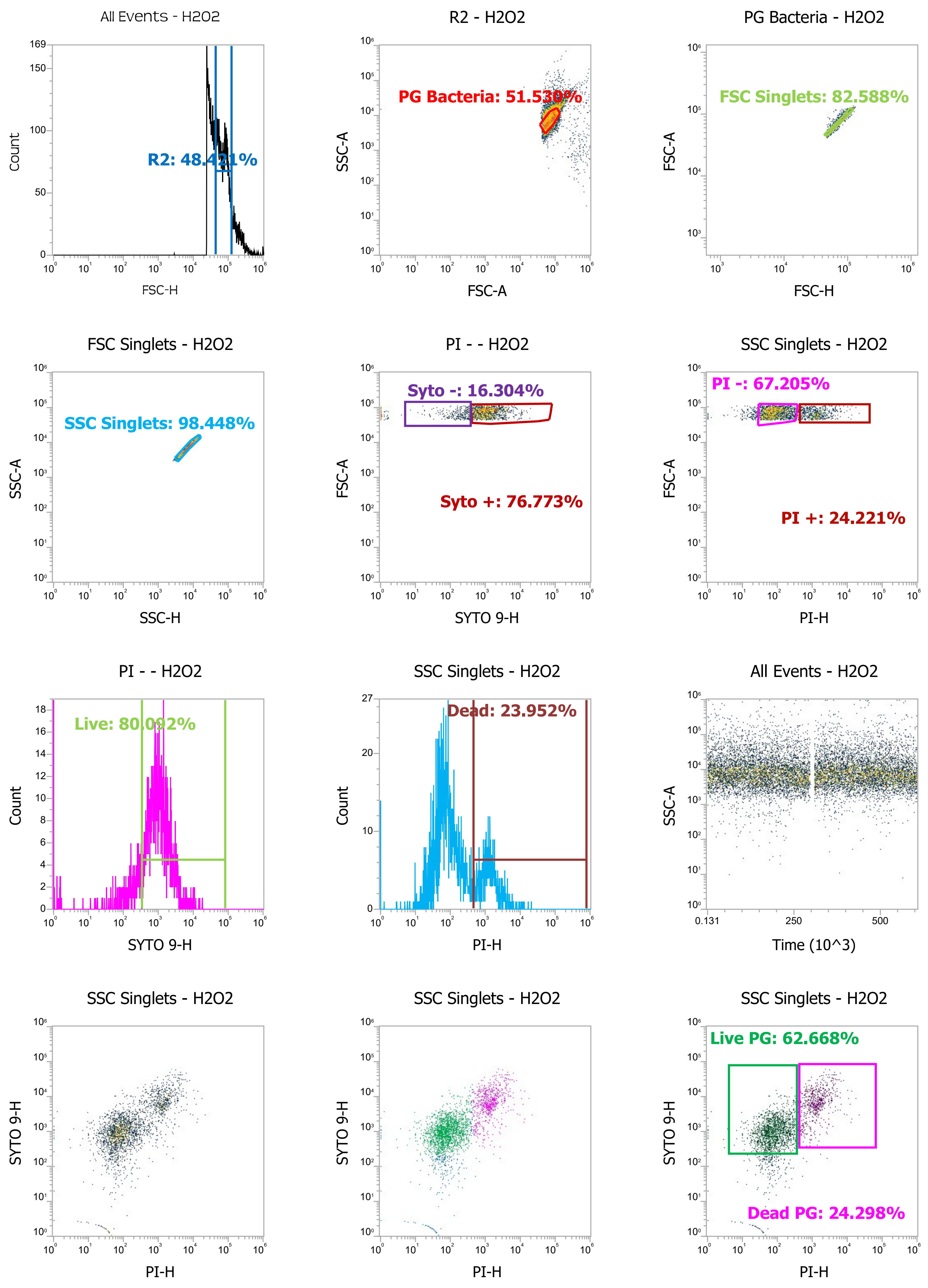

All Events - H2O2
R2: 48.421%
Count
FSC-H
R2 - H2O2
PG Bacteria: 51.530%
SSC-A
FSC-A
PG Bacteria - H2O2
FSC Singlets: 82.588%
FSC-A
FSC-H
FSC Singlets - H2O2
SSC Singlets: 98.448%
SSC-A
SSC-H
PI - - H2O2
Syto -: 16.304%
Syto +: 76.773%
FSC-A
SYTO 9-H
SSC Singlets - H2O2
PI -: 67.205%
PI +: 24.221%
FSC-A
PI-H
PI - - H2O2
Live: 80.092%
Count
SYTO 9-H
SSC Singlets - H2O2
Dead: 23.952%
Count
PI-H
All Events - H2O2
SSC-A
Time (10^3)
SSC Singlets - H2O2
SYTO 9-H
PI-H
SSC Singlets - H2O2
SYTO 9-H
PI-H
SSC Singlets - H2O2
Live PG: 62.668%
Dead PG: 24.298%
SYTO 9-H
PI-H

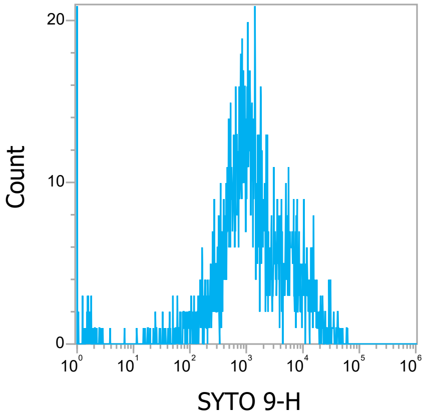

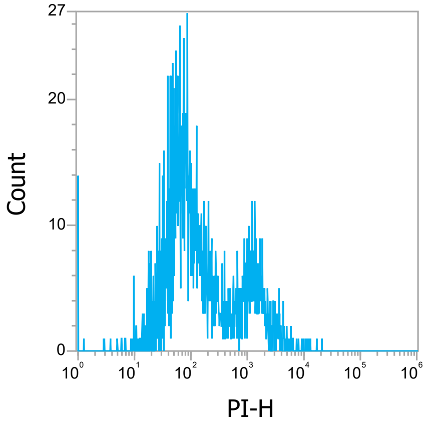

Experiment: **1st VBNC FC 4.2.24**
Group: **Group**
Sample: **H2O2**
Time Recorded: **12:57:47**

Name	Gate	Autogate Status	X Parameter	Y Parameter	Count	%Total	%Gated
All Events	All Events	N/A	N/A	N/A	12,821	100.000	100.000
R2	R2	N/A	FSC - FSC-H		6,208	48.421	48.421
PG Bacteria	PG Bacteria	N/A	FSC - FSC-A	SSC - SSC-A	3,199	24.951	51.530
FSC Singlets	FSC Singlets	N/A	FSC - FSC-H	FSC - FSC-A	2,642	20.607	82.588
SSC Singlets	SSC Singlets	N/A	SSC - SSC-H	SSC - SSC-A	2,601	20.287	98.448
PI -	PI -	N/A	PI - YL1-H	FSC - FSC-A	1,748	13.634	67.205
Syto +	Syto +	N/A	SYTO 9 - BL1-H	FSC - FSC-A	1,342	10.467	76.773
Syto -	Syto -	N/A	SYTO 9 - BL1-H	FSC - FSC-A	285	2.223	16.304
Live	Live	N/A	SYTO 9 - BL1-H		1,400	10.920	80.092
PI +	PI +	N/A	PI - YL1-H	FSC - FSC-A	630	4.914	24.221
Dead	Dead	N/A	PI - YL1-H		623	4.859	23.952
Live PG	Live PG	N/A	PI - YL1-H	SYTO 9 - BL1-H	1,630	12.714	62.668
Dead PG	Dead PG	N/A	PI - YL1-H	SYTO 9 - BL1-H	632	4.929	24.298

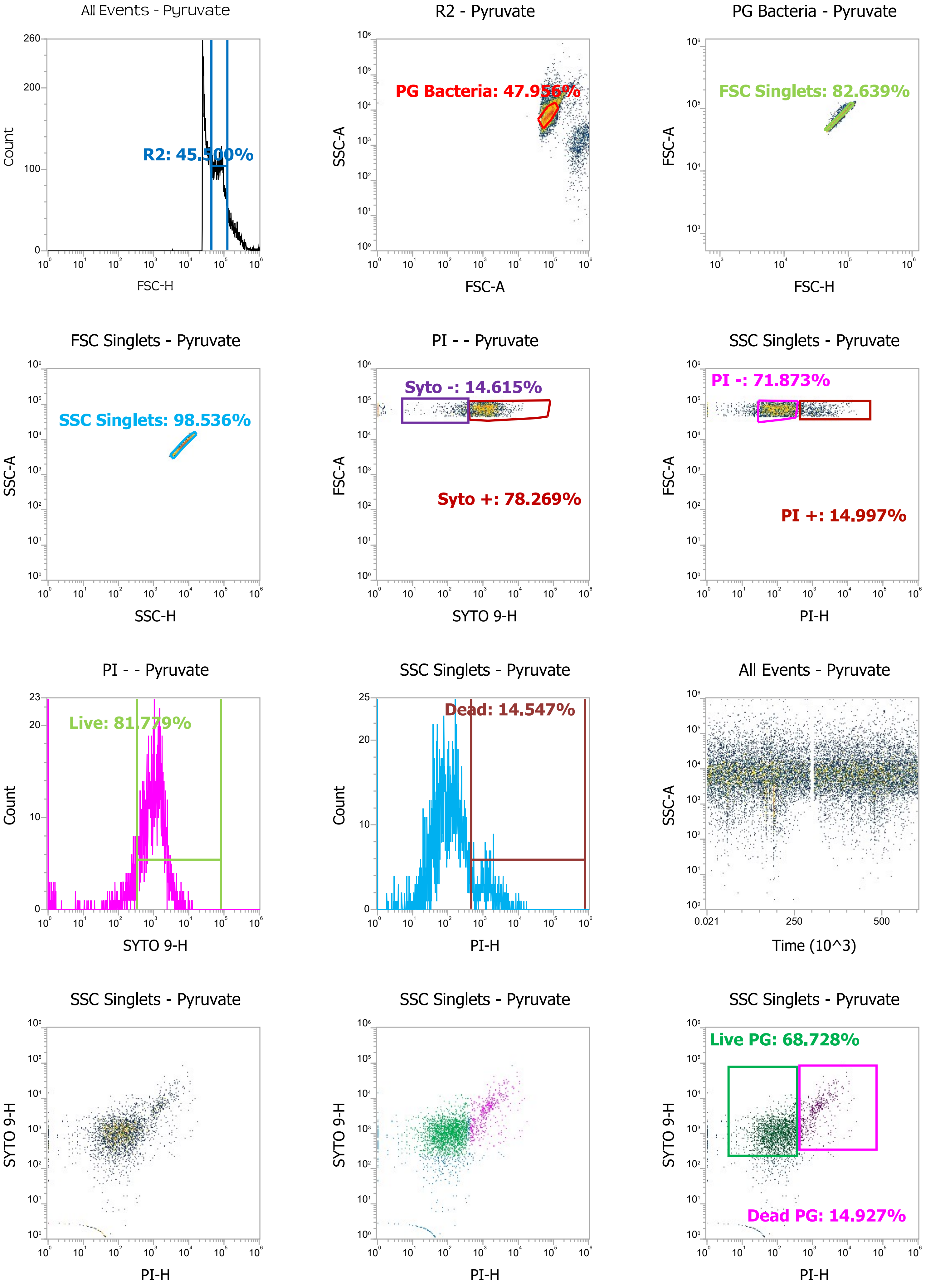
All Events - Pyruvate
Count
FSC-H
R2: 45.500%
R2 - Pyruvate
SSC-A
FSC-A
PG Bacteria: 47.956%
PG Bacteria - Pyruvate
FSC-A
FSC-H
FSC Singlets: 82.639%
FSC Singlets - Pyruvate
SSC-A
SSC-H
SSC Singlets: 98.536%
PI - - Pyruvate
FSC-A
SYTO 9-H
Syto -: 14.615%
Syto +: 78.269%
SSC Singlets - Pyruvate
FSC-A
PI-H
PI -: 71.873%
PI +: 14.997%
PI - - Pyruvate
Count
SYTO 9-H
Live: 81.779%
SSC Singlets - Pyruvate
Count
PI-H
Dead: 14.547%
All Events - Pyruvate
SSC-A
Time (10^3)
SSC Singlets - Pyruvate
SYTO 9-H
PI-H
SSC Singlets - Pyruvate
SYTO 9-H
PI-H
SSC Singlets - Pyruvate
SYTO 9-H
PI-H
Live PG: 68.728%
Dead PG: 14.927%

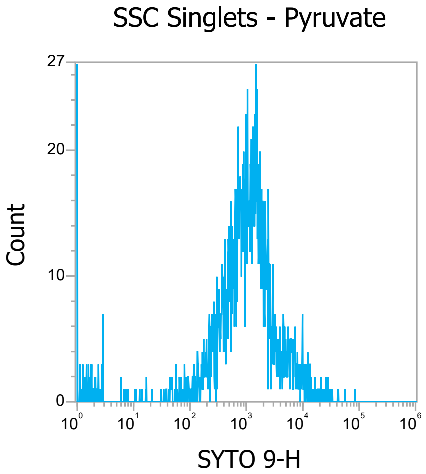

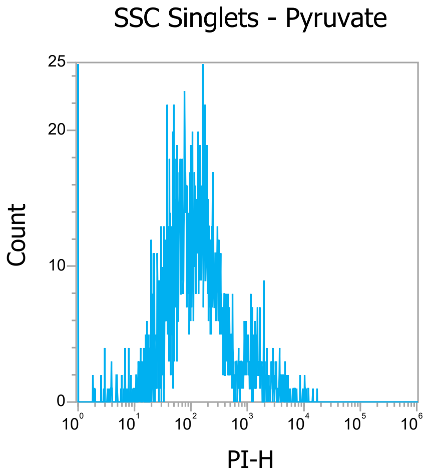

Experiment: **1st VBNC FC 4.2.24**
Group: **Group**
Sample: **Pyruvate**
Time Recorded: **13:13:18**

Name	Gate	Autogate Status	X Parameter	Y Parameter	Count	%Total	%Gated
All Events	All Events	N/A	N/A	N/A	16,288	100.000	100.000
R2	R2	N/A	FSC - FSC-H		7,411	45.500	45.500
PG Bacteria	PG Bacteria	N/A	FSC - FSC-A	SSC - SSC-A	3,554	21.820	47.956
FSC Singlets	FSC Singlets	N/A	FSC - FSC-H	FSC - FSC-A	2,937	18.032	82.639
SSC Singlets	SSC Singlets	N/A	SSC - SSC-H	SSC - SSC-A	2,894	17.768	98.536
PI -	PI -	N/A	PI - YL1-H	FSC - FSC-A	2,080	12.770	71.873
Syto +	Syto +	N/A	SYTO 9 - BL1-H	FSC - FSC-A	1,628	9.995	78.269
Syto -	Syto -	N/A	SYTO 9 - BL1-H	FSC - FSC-A	304	1.866	14.615
Live	Live	N/A	SYTO 9 - BL1-H		1,701	10.443	81.779
PI +	PI +	N/A	PI - YL1-H	FSC - FSC-A	434	2.665	14.997
Dead	Dead	N/A	PI - YL1-H		421	2.585	14.547
Live PG	Live PG	N/A	PI - YL1-H	SYTO 9 - BL1-H	1,989	12.211	68.728
Dead PG	Dead PG	N/A	PI - YL1-H	SYTO 9 - BL1-H	432	2.652	14.927

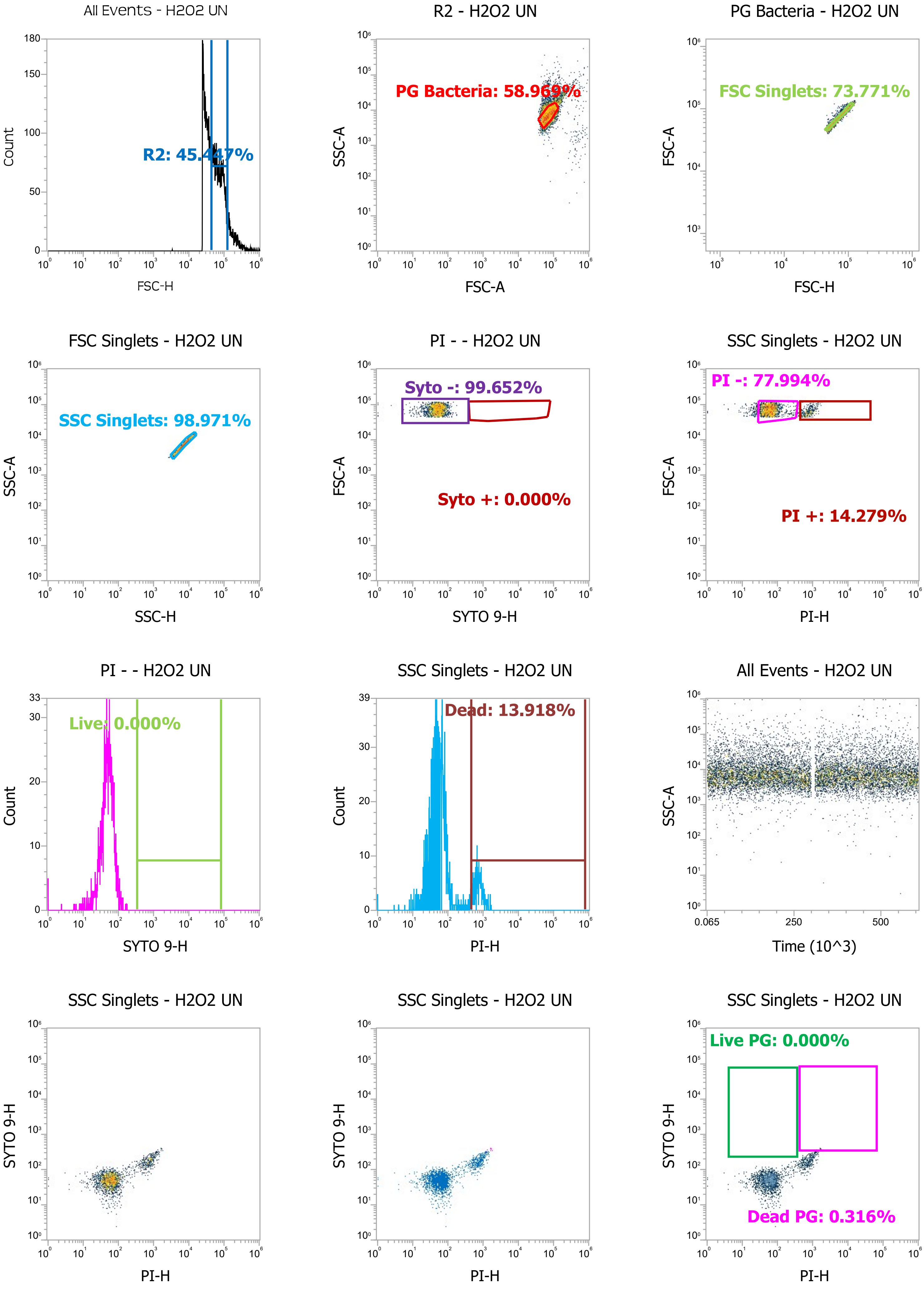
All Events - H2O2 UN
Count
FSC-H
R2: 45.447%
R2 - H2O2 UN
SSC-A
FSC-A
PG Bacteria: 58.969%
PG Bacteria - H2O2 UN
FSC-A
FSC-H
FSC Singlets: 73.771%
FSC Singlets - H2O2 UN
SSC-A
SSC-H
SSC Singlets: 98.971%
PI - - H2O2 UN
FSC-A
SYTO 9-H
Syto -: 99.652%
Syto +: 0.000%
SSC Singlets - H2O2 UN
FSC-A
PI-H
PI -: 77.994%
PI +: 14.279%
PI - - H2O2 UN
Count
SYTO 9-H
Live: 0.000%
SSC Singlets - H2O2 UN
Count
PI-H
Dead: 13.918%
All Events - H2O2 UN
SSC-A
Time (10^3)
SSC Singlets - H2O2 UN
SYTO 9-H
PI-H
SSC Singlets - H2O2 UN
SYTO 9-H
PI-H
SSC Singlets - H2O2 UN
SYTO 9-H
PI-H
Live PG: 0.000%
Dead PG: 0.316%

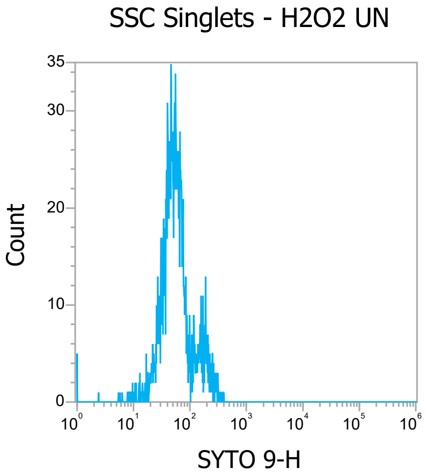

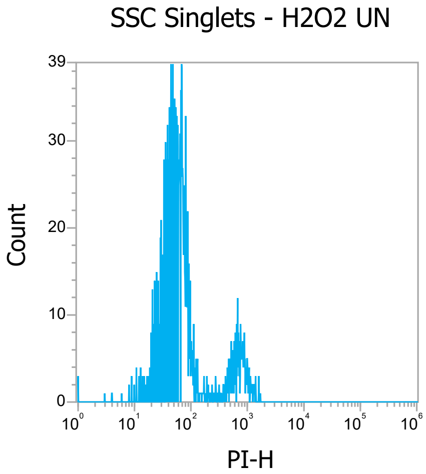

Experiment: **1st VBNC FC 4.2.24**
Group: **Group**
Sample: **H2O2 UN**
Time Recorded: **13:39:17**

Name	Gate	Autogate Status	X Parameter	Y Parameter	Count	%Total	%Gated
All Events	All Events	N/A	N/A	N/A	11,310	100.000	100.000
R2	R2	N/A	FSC - FSC-H		5,140	45.447	45.447
PG Bacteria	PG Bacteria	N/A	FSC - FSC-A	SSC - SSC-A	3,031	26.799	58.969
FSC Singlets	FSC Singlets	N/A	FSC - FSC-H	FSC - FSC-A	2,236	19.770	73.771
SSC Singlets	SSC Singlets	N/A	SSC - SSC-H	SSC - SSC-A	2,213	19.567	98.971
PI -	PI -	N/A	PI - YL1-H	FSC - FSC-A	1,726	15.261	77.994
Syto +	Syto +	N/A	SYTO 9 - BL1-H	FSC - FSC-A	0	0.000	0.000
Syto -	Syto -	N/A	SYTO 9 - BL1-H	FSC - FSC-A	1,720	15.208	99.652
Live	Live	N/A	SYTO 9 - BL1-H		0	0.000	0.000
PI +	PI +	N/A	PI - YL1-H	FSC - FSC-A	316	2.794	14.279
Dead	Dead	N/A	PI - YL1-H		308	2.723	13.918
Live PG	Live PG	N/A	PI - YL1-H	SYTO 9 - BL1-H	0	0.000	0.000
Dead PG	Dead PG	N/A	PI - YL1-H	SYTO 9 - BL1-H	7	0.062	0.316

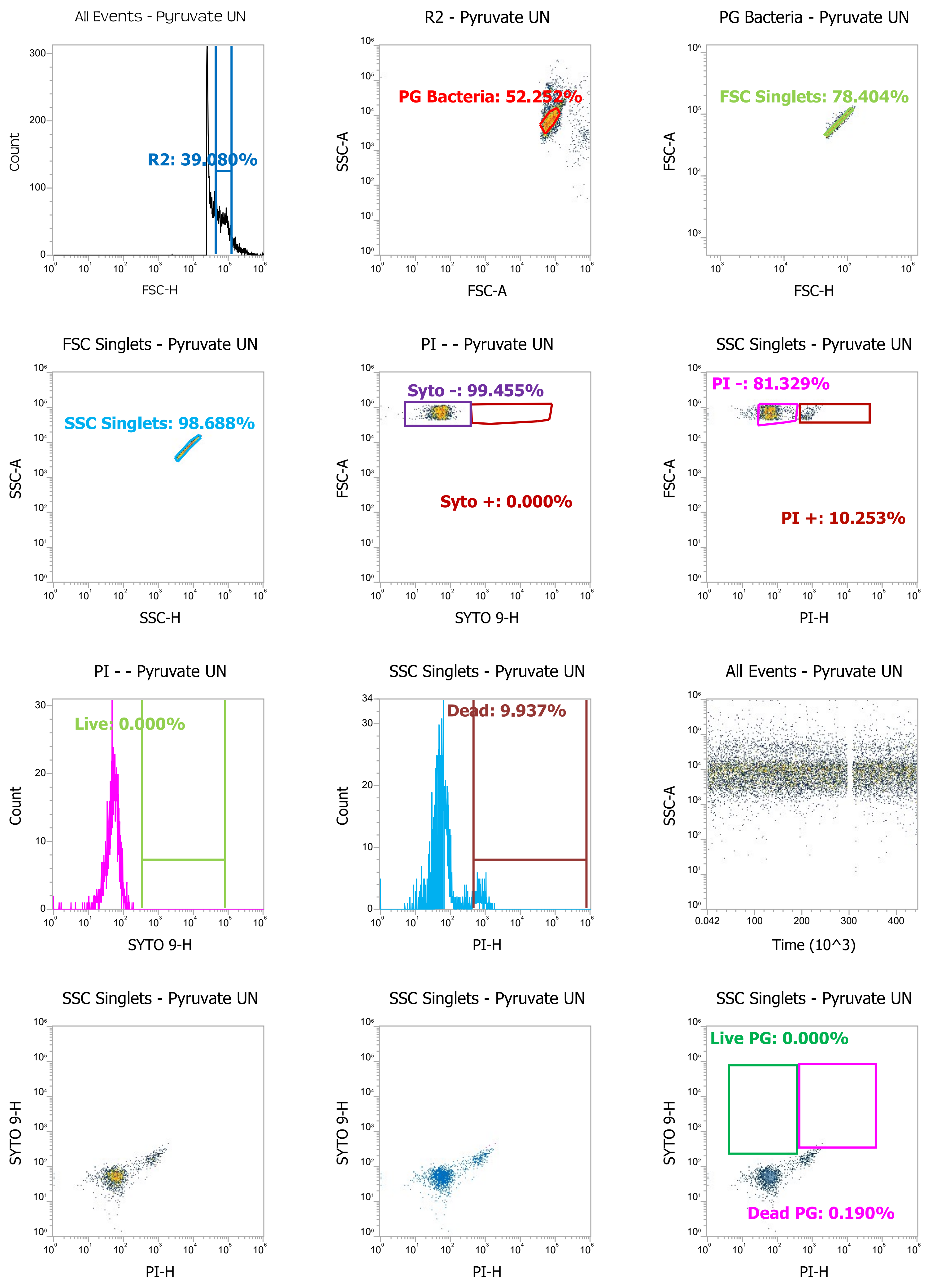
All Events - Pyruvate UN
R2: 39.080%
Count
FSC-H
R2 - Pyruvate UN
PG Bacteria: 52.252%
SSC-A
FSC-A
PG Bacteria - Pyruvate UN
FSC Singlets: 78.404%
FSC-A
FSC-H
FSC Singlets - Pyruvate UN
SSC Singlets: 98.688%
SSC-A
SSC-H
PI - - Pyruvate UN
Syto -: 99.455%
Syto +: 0.000%
FSC-A
SYTO 9-H
SSC Singlets - Pyruvate UN
PI -: 81.329%
PI +: 10.253%
FSC-A
PI-H
PI - - Pyruvate UN
Live: 0.000%
Count
SYTO 9-H
SSC Singlets - Pyruvate UN
Dead: 9.937%
Count
PI-H
All Events - Pyruvate UN
SSC-A
Time (10^3)
SSC Singlets - Pyruvate UN
SYTO 9-H
PI-H
SSC Singlets - Pyruvate UN
SYTO 9-H
PI-H
SSC Singlets - Pyruvate UN
Live PG: 0.000%
Dead PG: 0.190%
SYTO 9-H
PI-H

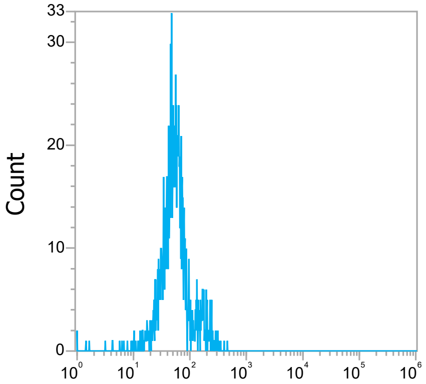

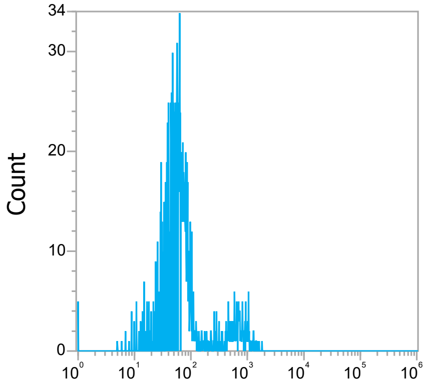

Experiment: **1st VBNC FC 4.2.24**
Group: **Group**
Sample: **Pyruvate UN**
Time Recorded: **13:51:39**

Name	Gate	Autogate Status	X Parameter	Y Parameter	Count	%Total	%Gated
All Events	All Events	N/A	N/A	N/A	10,000	100.000	100.000
R2	R2	N/A	FSC - FSC-H		3,908	39.080	39.080
PG Bacteria	PG Bacteria	N/A	FSC - FSC-A	SSC - SSC-A	2,042	20.420	52.252
FSC Singlets	FSC Singlets	N/A	FSC - FSC-H	FSC - FSC-A	1,601	16.010	78.404
SSC Singlets	SSC Singlets	N/A	SSC - SSC-H	SSC - SSC-A	1,580	15.800	98.688
PI -	PI -	N/A	PI - YL1-H	FSC - FSC-A	1,285	12.850	81.329
Syto +	Syto +	N/A	SYTO 9 - BL1-H	FSC - FSC-A	0	0.000	0.000
Syto -	Syto -	N/A	SYTO 9 - BL1-H	FSC - FSC-A	1,278	12.780	99.455
Live	Live	N/A	SYTO 9 - BL1-H		0	0.000	0.000
PI +	PI +	N/A	PI - YL1-H	FSC - FSC-A	162	1.620	10.253
Dead	Dead	N/A	PI - YL1-H		157	1.570	9.937
Live PG	Live PG	N/A	PI - YL1-H	SYTO 9 - BL1-H	0	0.000	0.000
Dead PG	Dead PG	N/A	PI - YL1-H	SYTO 9 - BL1-H	3	0.030	0.190

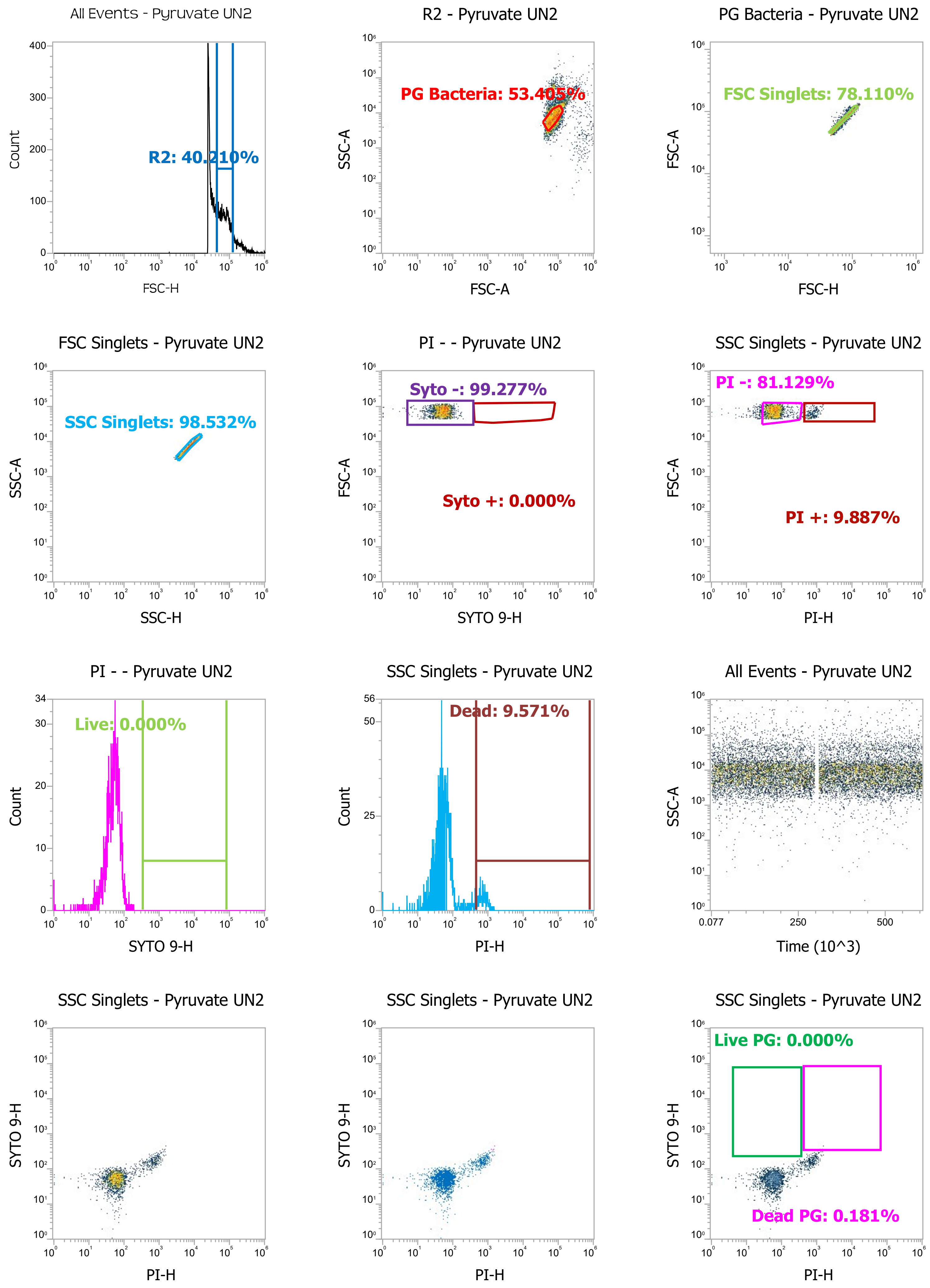
All Events - Pyruvate UN2
R2: 40.210%
Count
FSC-H
R2 - Pyruvate UN2
PG Bacteria: 53.405%
SSC-A
FSC-A
PG Bacteria - Pyruvate UN2
FSC Singlets: 78.110%
FSC-A
FSC-H
FSC Singlets - Pyruvate UN2
SSC Singlets: 98.532%
SSC-A
SSC-H
PI - - Pyruvate UN2
Syto -: 99.277%
Syto +: 0.000%
FSC-A
SYTO 9-H
SSC Singlets - Pyruvate UN2
PI -: 81.129%
PI +: 9.887%
FSC-A
PI-H
PI - - Pyruvate UN2
Live: 0.000%
Count
SYTO 9-H
SSC Singlets - Pyruvate UN2
Dead: 9.571%
Count
PI-H
All Events - Pyruvate UN2
SSC-A
Time (10^3)
SSC Singlets - Pyruvate UN2
SYTO 9-H
PI-H
SSC Singlets - Pyruvate UN2
SYTO 9-H
PI-H
SSC Singlets - Pyruvate UN2
Live PG: 0.000%
Dead PG: 0.181%
SYTO 9-H
PI-H

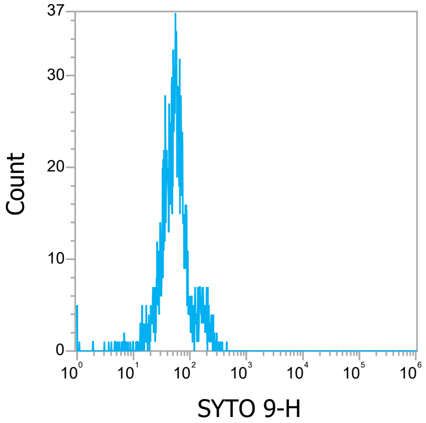

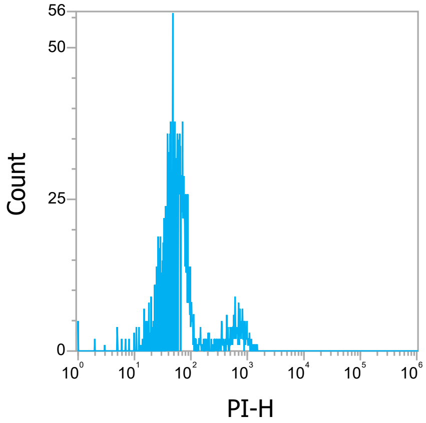

Experiment: **1st VBNC FC 4.2.24**
Group: **Group**
Sample: **Pyruvate UN2**
Time Recorded: **14:09:11**

Name	Gate	Autogate Status	X Parameter	Y Parameter	Count	%Total	%Gated
All Events	All Events	N/A	N/A	N/A	13,402	100.000	100.000
R2	R2	N/A	FSC - FSC-H		5,389	40.210	40.210
PG Bacteria	PG Bacteria	N/A	FSC - FSC-A	SSC - SSC-A	2,878	21.474	53.405
FSC Singlets	FSC Singlets	N/A	FSC - FSC-H	FSC - FSC-A	2,248	16.774	78.110
SSC Singlets	SSC Singlets	N/A	SSC - SSC-H	SSC - SSC-A	2,215	16.527	98.532
PI -	PI -	N/A	PI - YL1-H	FSC - FSC-A	1,797	13.408	81.129
Syto +	Syto +	N/A	SYTO 9 - BL1-H	FSC - FSC-A	0	0.000	0.000
Syto -	Syto -	N/A	SYTO 9 - BL1-H	FSC - FSC-A	1,784	13.311	99.277
Live	Live	N/A	SYTO 9 - BL1-H		0	0.000	0.000
PI +	PI +	N/A	PI - YL1-H	FSC - FSC-A	219	1.634	9.887
Dead	Dead	N/A	PI - YL1-H		212	1.582	9.571
Live PG	Live PG	N/A	PI - YL1-H	SYTO 9 - BL1-H	0	0.000	0.000
Dead PG	Dead PG	N/A	PI - YL1-H	SYTO 9 - BL1-H	4	0.030	0.181

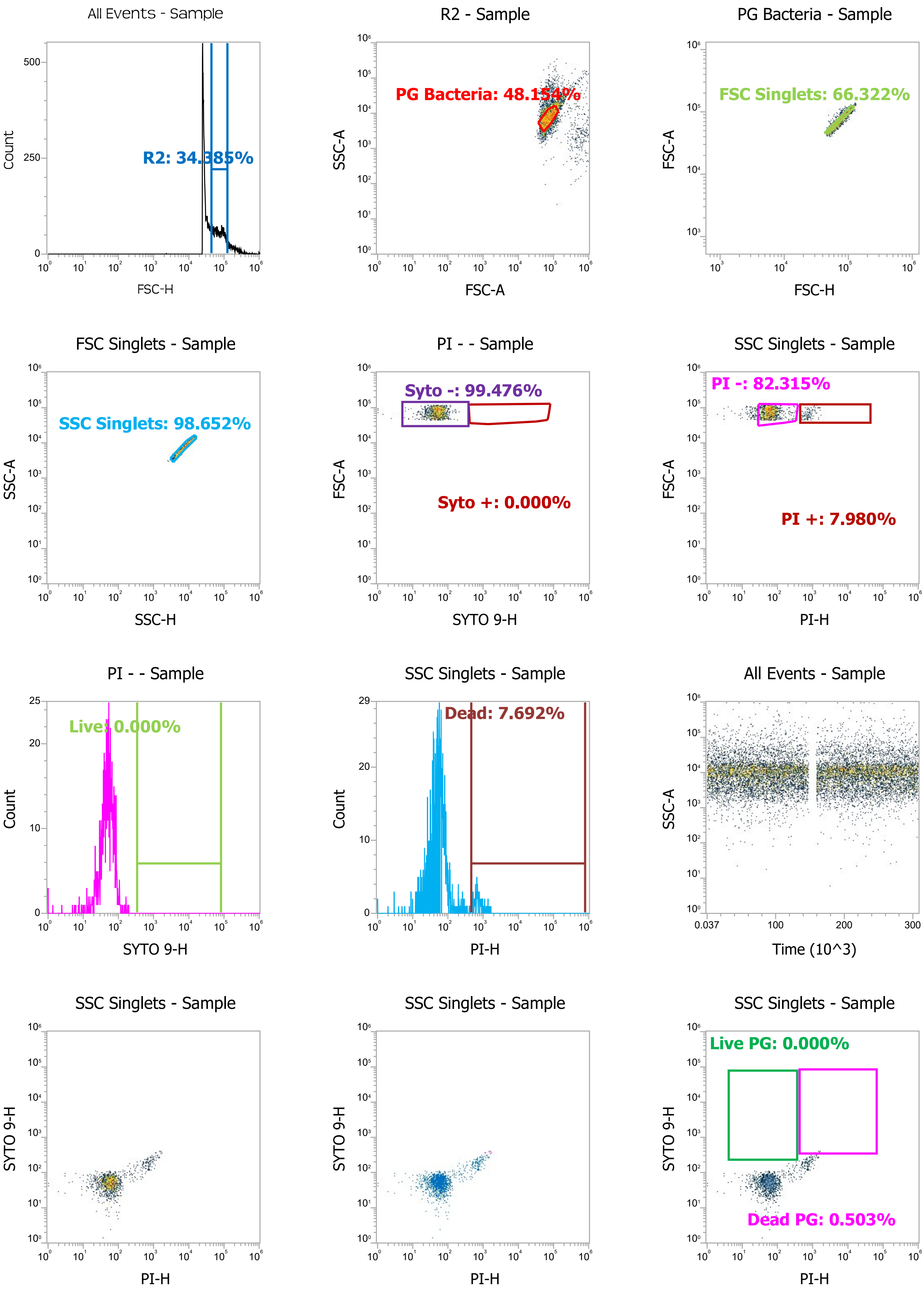

All Events - Sample
R2: 34.385%
Count
FSC-H
R2 - Sample
PG Bacteria: 48.154%
SSC-A
FSC-A
PG Bacteria - Sample
FSC Singlets: 66.322%
FSC-A
FSC-H
FSC Singlets - Sample
SSC Singlets: 98.652%
SSC-A
SSC-H
PI - - Sample
Syto -: 99.476%
Syto +: 0.000%
FSC-A
SYTO 9-H
SSC Singlets - Sample
PI -: 82.315%
PI +: 7.980%
FSC-A
PI-H
PI - - Sample
Live: 0.000%
Count
SYTO 9-H
SSC Singlets - Sample
Dead: 7.692%
Count
PI-H
All Events - Sample
SSC-A
Time (10^3)
SSC Singlets - Sample
SYTO 9-H
PI-H
SSC Singlets - Sample
SYTO 9-H
PI-H
SSC Singlets - Sample
Live PG: 0.000%
Dead PG: 0.503%
SYTO 9-H
PI-H

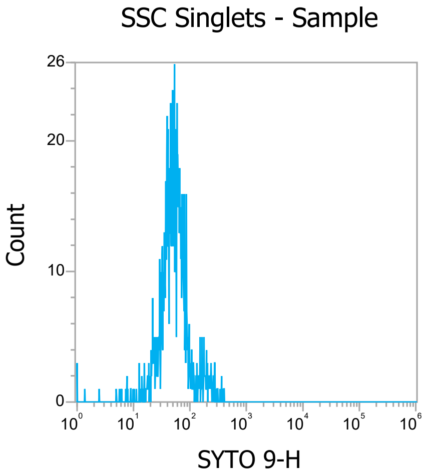

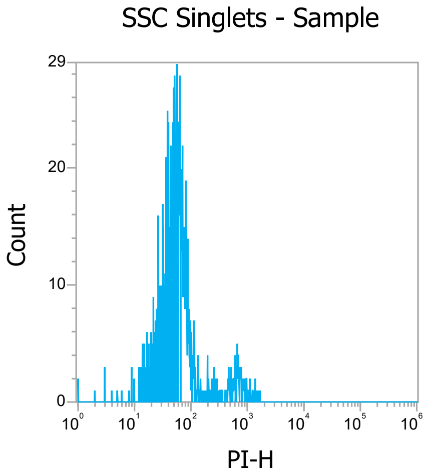

Experiment: **1st VBNC FC 4.2.24**
Group: **Group**
Sample: **Sample**
Time Recorded: **14:15:28**

Name	Gate	Autogate Status	X Parameter	Y Parameter	Count	%Total	%Gated
All Events	All Events	N/A	N/A	N/A	12,840	100.000	100.000
R2	R2	N/A	FSC - FSC-H		4,415	34.385	34.385
PG Bacteria	PG Bacteria	N/A	FSC - FSC-A	SSC - SSC-A	2,126	16.558	48.154
FSC Singlets	FSC Singlets	N/A	FSC - FSC-H	FSC - FSC-A	1,410	10.981	66.322
SSC Singlets	SSC Singlets	N/A	SSC - SSC-H	SSC - SSC-A	1,391	10.833	98.652
PI -	PI -	N/A	PI - YL1-H	FSC - FSC-A	1,145	8.917	82.315
Syto +	Syto +	N/A	SYTO 9 - BL1-H	FSC - FSC-A	0	0.000	0.000
Syto -	Syto -	N/A	SYTO 9 - BL1-H	FSC - FSC-A	1,139	8.871	99.476
Live	Live	N/A	SYTO 9 - BL1-H		0	0.000	0.000
PI +	PI +	N/A	PI - YL1-H	FSC - FSC-A	111	0.864	7.980
Dead	Dead	N/A	PI - YL1-H		107	0.833	7.692
Live PG	Live PG	N/A	PI - YL1-H	SYTO 9 - BL1-H	0	0.000	0.000
Dead PG	Dead PG	N/A	PI - YL1-H	SYTO 9 - BL1-H	7	0.055	0.503

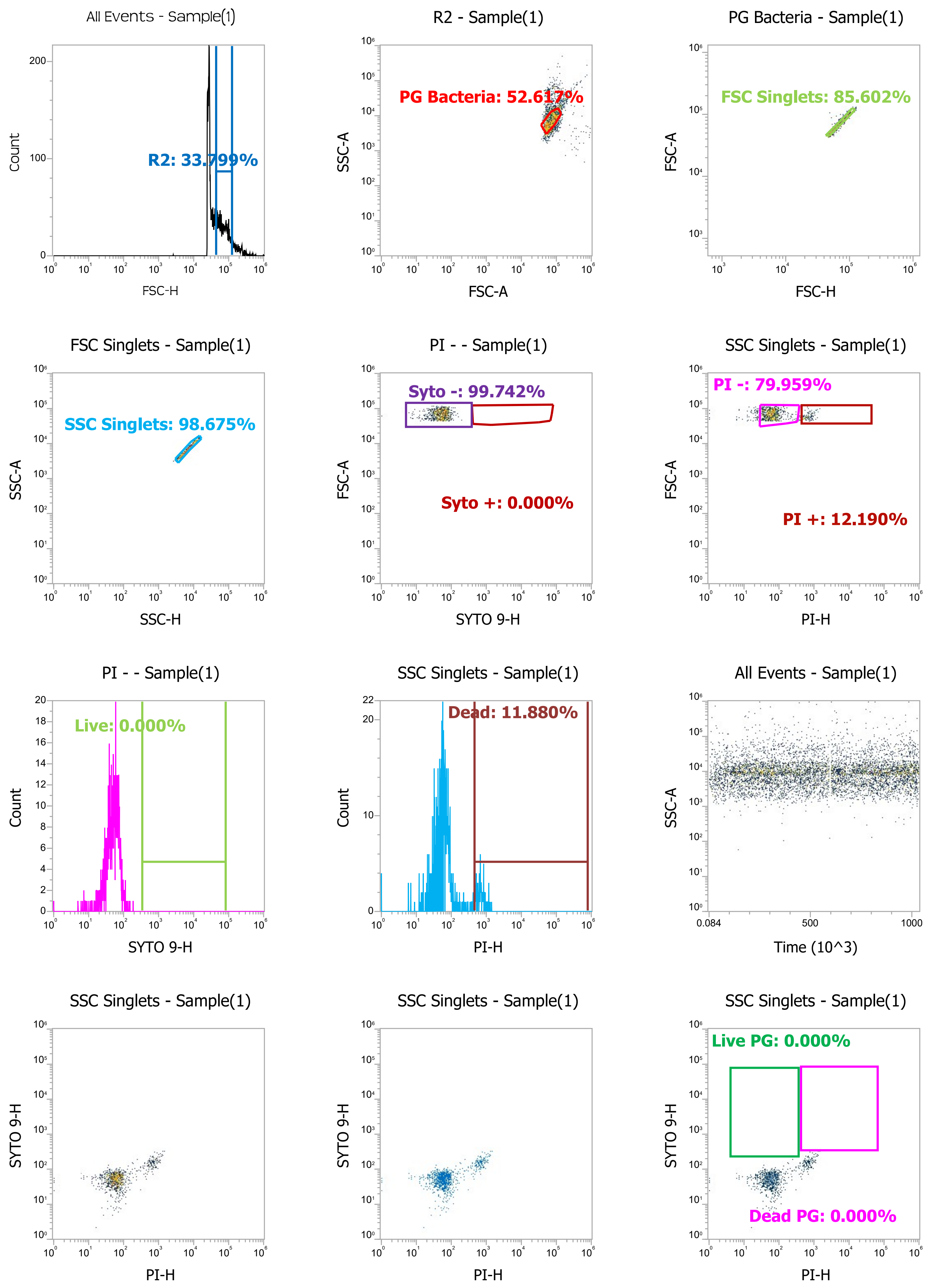
All Events - Sample(1)
R2: 33.799%
Count
FSC-H
R2 - Sample(1)
PG Bacteria: 52.617%
SSC-A
FSC-A
PG Bacteria - Sample(1)
FSC Singlets: 85.602%
FSC-A
FSC-H
FSC Singlets - Sample(1)
SSC Singlets: 98.675%
SSC-A
SSC-H
PI - - Sample(1)
Syto -: 99.742%
Syto +: 0.000%
FSC-A
SYTO 9-H
SSC Singlets - Sample(1)
PI -: 79.959%
PI +: 12.190%
FSC-A
PI-H
PI - - Sample(1)
Live: 0.000%
Count
SYTO 9-H
SSC Singlets - Sample(1)
Dead: 11.880%
Count
PI-H
All Events - Sample(1)
SSC-A
Time (10^3)
SSC Singlets - Sample(1)
SYTO 9-H
PI-H
SSC Singlets - Sample(1)
SYTO 9-H
PI-H
SSC Singlets - Sample(1)
Live PG: 0.000%
Dead PG: 0.000%
SYTO 9-H
PI-H

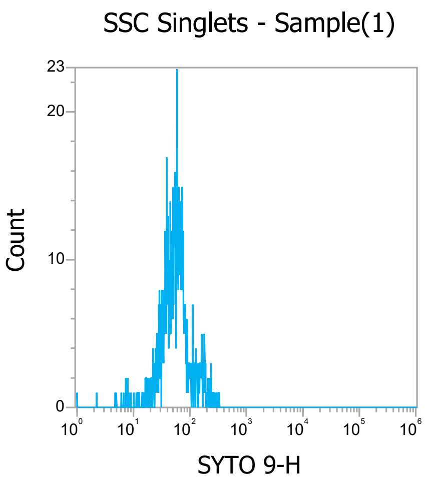

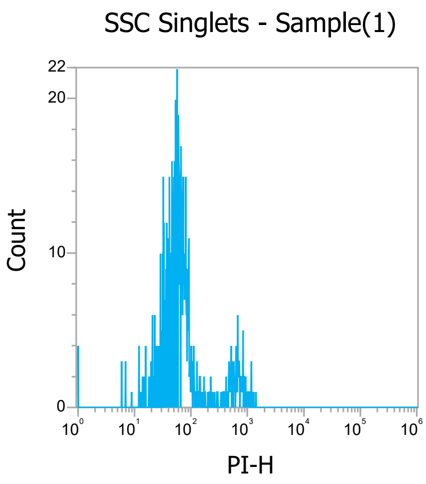

Experiment: **1st VBNC FC 4.2.24**
Group: **Group**
Sample: **Sample(1)**
Time Recorded: **14:36:00**

Name	Gate	Autogate Status	X Parameter	Y Parameter	Count	%Total	%Gated
All Events	All Events	N/A	N/A	N/A	6,444	100.000	100.000
R2	R2	N/A	FSC - FSC-H		2,178	33.799	33.799
PG Bacteria	PG Bacteria	N/A	FSC - FSC-A	SSC - SSC-A	1,146	17.784	52.617
FSC Singlets	FSC Singlets	N/A	FSC - FSC-H	FSC - FSC-A	981	15.223	85.602
SSC Singlets	SSC Singlets	N/A	SSC - SSC-H	SSC - SSC-A	968	15.022	98.675
PI -	PI -	N/A	PI - YL1-H	FSC - FSC-A	774	12.011	79.959
Syto +	Syto +	N/A	SYTO 9 - BL1-H	FSC - FSC-A	0	0.000	0.000
Syto -	Syto -	N/A	SYTO 9 - BL1-H	FSC - FSC-A	772	11.980	99.742
Live	Live	N/A	SYTO 9 - BL1-H		0	0.000	0.000
PI +	PI +	N/A	PI - YL1-H	FSC - FSC-A	118	1.831	12.190
Dead	Dead	N/A	PI - YL1-H		115	1.785	11.880
Live PG	Live PG	N/A	PI - YL1-H	SYTO 9 - BL1-H	0	0.000	0.000
Dead PG	Dead PG	N/A	PI - YL1-H	SYTO 9 - BL1-H	0	0.000	0.000
